# Hyperosmolarity impedes the cross-priming competence of dendritic cells in a TRIF-dependent manner

**DOI:** 10.1038/s41598-017-00434-y

**Published:** 2017-03-22

**Authors:** Zoran V. Popovic, Maria Embgenbroich, Federica Chessa, Viola Nordström, Mahnaz Bonrouhi, Thomas Hielscher, Norbert Gretz, Shijun Wang, Daniel Mathow, Thomas Quast, Jan-Gero Schloetel, Waldemar Kolanus, Sven Burgdorf, Hermann-Josef Gröne

**Affiliations:** 1 Department of Cellular and Molecular Pathology, German Cancer Research Center, Heidelberg, Germany; 2 Department of Biostatistics, German Cancer Research Center, Heidelberg, Germany; 3 Institute of Pathology, University Hospital Mannheim, University of Heidelberg, Mannheim, Germany; 4 Medical Research Center, University Hospital Mannheim, University of Heidelberg, Mannheim, Germany; 5 Department of Cellular Immunology, LIMES Institute, University of Bonn, Bonn, Germany; 6 Department of Molecular Immunology and Cell Biology, LIMES Institute, University of Bonn, Bonn, Germany; 7 Department of Membrane Biochemistry, LIMES Institute, University of Bonn, Bonn, Germany

## Abstract

Tissue osmolarity varies among different organs and can be considerably increased under pathologic conditions. Hyperosmolarity has been associated with altered stimulatory properties of immune cells, especially macrophages and dendritic cells. We have recently reported that dendritic cells upon exposure to hypertonic stimuli shift their profile towards a macrophage-M2-like phenotype, resulting in attenuated local alloreactivity during acute kidney graft rejection. Here, we examined how hyperosmotic microenvironment affects the cross-priming capacity of dendritic cells. Using ovalbumin as model antigen, we showed that exposure of dendritic cells to hyperosmolarity strongly inhibits activation of antigen-specific T cells despite enhancement of antigen uptake, processing and presentation. We identified TRIF as key mediator of this phenomenon. Moreover, we detected a hyperosmolarity-triggered, TRIF-dependent clustering of MHCI loaded with the ovalbumin-derived epitope, but not of overall MHCI molecules, providing a possible explanation for a reduced T cell activation. Our findings identify dendritic cells as important players in hyperosmolarity-mediated immune imbalance and provide evidence for a novel pathway of inhibition of antigen specific CD8^+^ T cell response in a hypertonic micromilieu.

## Introduction

MHCI-mediated antigen presentation is essential for an effective cytotoxic immune response against infected cells and tumor cells. Specific subsets of mononuclear phagocytes (MoPh) are known to be capable of presenting exogenous antigens on MHCI molecules (cross-presentation). This feature was originally assigned to CD8α^+^ dendritic cells (DCs) in mouse/BDCA3^+^ and BDCA1^+^ DCs in man^1, 2^. In the ‘endosome-to cytosol’cross-presentation pathway, ingested antigens are translocated from endosomes into the cytosol and degraded by the proteasome. Resulting peptides are re-translocated into endosomes or the endoplasmic reticulum (ER) for loading on MHCI molecules^3–6^. Translocation of the antigen from endosomes to the cytosol is shown to be enhanced upon endotoxin stimulation and is considered to be mediated by the trimeric translocon complex Sec61, which normally enables protein transport in and out of the ER but which is translocated toward antigen-containing endosomes upon TRIF signaling^7–10^. The efficacy of cross-presentation seems to be modulated by a variety of conditions, such as type of antigen, DC activation status, specific tissue environment and inflammatory stimuli^11, 12^. It is still not known which microenvironmental signals may influence antigen processing and presentation by DC. The micromilieu includes biophysical factors such as osmolarity; mainly due to technical challenges in measuring biophysical parameters in interstitial space, their role in MoPh activation remains largely unexplored. Physiologically hyperosmolar interstitial milieus of renal medulla, lymphoid tissue compartments or intervertebral discs as well as hyperosmolar tumor tissue may shape the pattern of immune response^13–19^. It has been reported that macrophages (Mϕ) recognize NaCl hypertonicity as a chemotactic stimulus and migrate in the direction of excess salt concentration, independent of NFAT5 activation^20^. Others have shown that increased Na^+^ concentration may affect M1-, M2- and inflammasome-activated Mϕ via a complex microenvironmental set of signals and varying mechanisms^14, 21–24^. Regarding the influence of hyperosmotic pressure on DC function, we have provided evidence that a NaCl-hyperosmolar micromilieu may switch a classical DC signature towards a Mϕ M2-like pattern, leading to a lower alloreactivity of renal medullary DCs in a murine renal transplantation model^14^. Here, we have investigated the impact of hyperosmolarity on activation of CD8^+^ T cells. We have detected that NaCl-hypertonic stress in both immunologically silent and pro-inflammatory micromilieus strongly inhibits the capacity of dendritic cells to activate CD8^+^ T cells in a TRIF-dependent, NFAT5-independent manner. This effect is potentially tuned by a complex set of events which result in surface MHCI-antigen cluster formation.

## Results

### Hyperosmolarity inhibits activation of CD8^+^ T cells by dendritic cells in a NFAT5-independent manner

**Figure 1:**
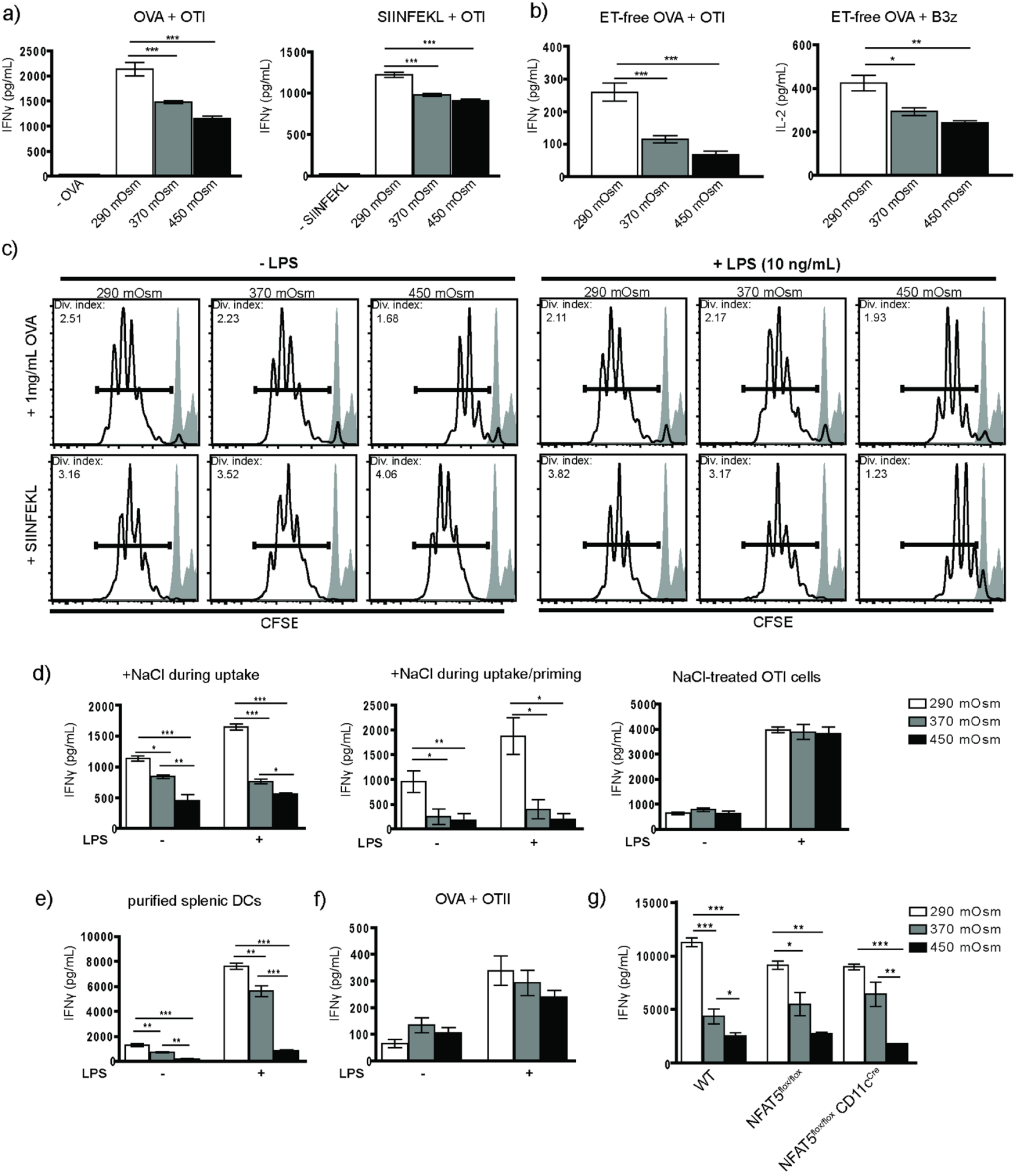
Hyperosmotic microenvironment inhibits CD8^+^ T cell activation. (**a**) BMDCs raised in isotonic or hypersomotic conditions (290 mOsm, 370 mOsm and 450 mOsm) were incubated with OVA (0,5 mg/mL; left graph, n = 8) or SIINFEKL peptide (1 µg/mL, right graph, n = 4), without or with LPS (10 ng/mL) for 2 hours, followed by overnight (17 hours) co-incubation with OTI cells. (**b**) Using the same approach, OVA was substituted by endotoxin-free OVA (1 mg/mL, left graph, n = 8), and B3z cells were used instead of OTI cells (right graph, n = 4). (**c**) CFSE-labeled OTI cells were incubated with OVA-primed BMDCs for 96 hours and analyzed by flow cytometry for CFSE dilution as a direct measure of OTI proliferation (representative histograms, n = 4). (**d**) BMDCs developed in isotonic conditions were exposed to normosmolar or indicated hyperosmolar microenvironment during ET-free OVA uptake (left graph, 2 hours; n = 4); continuously during ET-free OVA uptake and co-incubation with OTI cells (middle graph, n = 4) and used in T cell activation assay as described. In a separate experiment, solely OTI cells were treated with NaCl-rich medium for 2 hours upon sorting and used in the BMDC/OVA assay as described (right graph, n = 4). (**e**) Purified CD11c^+^ dendritic cells gained by magnetic separation from spleens of C57BL/6 mice were exposed to isotonic or NaCl-enriched media for a period of 6 hours and used in ET-free OVA/OTI T cell activation assay (n = 4). (**f**) OTII cells were co-incubated with ET-free OVA-loaded BMDCs from different osmolarities, without or with (10 ng/mL) LPS stimulus (n = 5). (**g**) BMDCs derived from bone marrows of CD11c-cell specific NFAT5-deficient mice were developed in isotonic or NaCl-hypertonic conditions and used in the cross-priming OVA/OTI assay as previously described (n = 4). IFN-γ and IL-2 were measured from cell culture supernatants. Statistical data are expressed as mean ± SEM and are representative of at least two independent experiments (*p ≤ 0.05, **p ≤ 0.01, ***p ≤ 0.001).

To investigate the functional effect of hyperosmotic stress on CD8^+^ T cell activation, bone marrow derived dendritic cells (BMDCs) were exposed to high salt (370 mOsm, 450 mOsm) continuously during the last four days of GM-CSF-mediated differentiation. On day 7, the hypertonic medium was replaced by isotonic to avoid biochemical or biophysical interference of NaCl with cellular or molecular components applied in further experimental steps. The cells were further exposed to OVA either in endotoxin-free conditions or upon pulsing with LPS and used in priming assays. Exposure of BMDCs to hyperosmolarity during development resulted in diminished T cell activation in comparison to BMDCs from isotonic medium, independent of the source of OVA (Fig. 1a,b; Suppl. Fig. 1a,b). A comparable effect of high salt medium was observed in BMDCs pulsed with SIINFEKL peptide instead of OVA (Fig. 1a). Concordant to previously published data^14^, we have not detected ultrastructural hallmarks of cell death in BMDCs raised in hyperosmolarity (Suppl. Fig. 2a). Already ‘moderately’ hyperosmotic medium (340 mOsm) could trigger a significant reduction of CD8^+^ T cell activation (Suppl. Fig. 2b). Direct measurement of OTI cell proliferation went in line with cytokine secretion data (Fig. 1c), Interestingly, significant alterations in secretion of IFNγ were already observed at 370 mOsm, whereas clear differences in proliferation were only seen at 450 mOsm, pointing out that cytokine secretion might be more sensitive to the hypertonic conditions of the DCs than OTI proliferation. To examine the short-term effects of NaCl-enriched medium on BMDCs differentiated in isotonic conditions, the cells were exposed to high salt solely during OVA uptake, or alternatively during both OVA uptake and co-culture with OTI cells; in both cases a significant inhibition of cross-priming was evident (Fig. 1d), suggesting that long-term exposure of immature BMDCs is not necessary for its blocking effect on cross-priming. Interestingly, the negative effects on T cell priming seemed to be enhanced when NaCl was still present during T cell activation. To examine if T cell activity might be as well compromised under conditions of hyperosmolarity, we exposed OTI cells to hypertonic medium 2 hours prior to adding them to OVA-loaded BMDCs raised in normosmotic (290 mOsm) conditions. We could not detect a significant effect of NaCl on the activity of OTI cells (Fig. 1d). In addition, *ex vivo* dendritic cells sorted from spleen have been exposed to hypertonic conditions and used in the cross-priming assay; the effect of NaCl resembled the data obtained by BMDCs (Fig. 1e). This indicates that our *in vitro* data seem to reflect an *in vivo* setting and that an evident heterogeneity of ‘BMDCs’ (BM-MoPh) likely did not affect our results. The MHC class II mediated antigen presentation by BMDCs was not significantly altered (Fig. 1f). We have further tested if a biologically inert, nonionic osmotic reagent can cause a similar effect on priming of OTI cells; application of mannitol (450 mOsm) resulted in a less pronounced, albeit statistically significant inhibition of cross-priming (Suppl. Fig. 1c). BMDCs of both NFAT5^flox/flox^CD11c^Cre^ (Fig. 1g) and NFAT5^−/−^ (Suppl. Fig. 1d) origin displayed similar tendency, demonstrating that the influence of hyperosmolarity on cross-priming does not depend on NFAT5, a transcription factor previously shown to regulate specific immunomodulatory effects of NaCl.

### Hypertonic microenvironment enhances uptake, processing and cross-presentation of OVA by BMDCs

**Figure 2:**
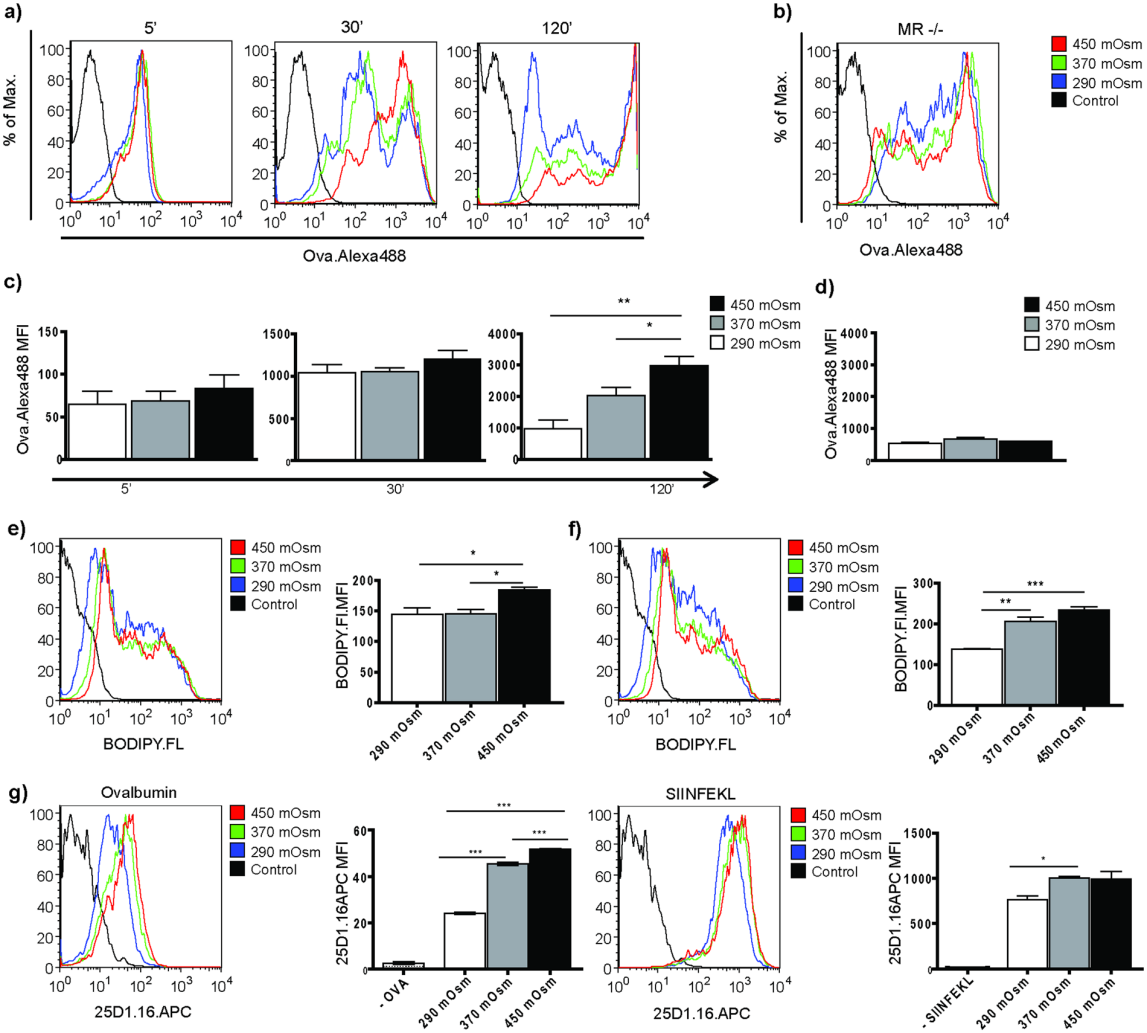
NaCl-rich micromilieu enhances uptake, processing and cross-presentation of ovalbumin. (**a**,**b**) Representative histograms showing time kinetics (5 min, 30 min, 120 min) of OVA.Alexa488 (1 µg/mL) uptake by BMDCs developed from C57BL/6 mice (**a**) or MR^−/−^ mice (**b**) in NaCl-hypertonic conditions (control = autofluorescence of BMDCs in FL1 channel; (**b**) representative histogram plot showing the 120 min time point). (**c**,**d**) Statistical graphs displaying mean fluorescence intensity of samples from (**a,b**), respectively (n = 5). (**e**,**f**) Representative histogram plots (left) and statistical graphs (right) showing BODIPY.FL mean fluorescence intensity upon uptake and degradation of DQ-OVA (10 µg/mL, 4 hours) by BMDCs of WT C57BL/6 (**e**) and MR^−/−^ (**f**) origin, developed in NaCl-hypertonic conditions as indicated. (n = 4) (**g**) BMDCs of WT origin developed in isotonic or NaCl-hypertonic media were incubated either with OVA (2 mg/mL, left) or (**h**) SIINFEKL peptide (1 µg/mL, right) for 17 hours (overnight). Expression of SIINFEKL bound to H-2Kb of MHC class I on the surface of BMDCs was evaluated using 25D1.16.APC antibody, here displayed as representative histograms (left) and statistical graphs (right). Statistical data are expressed as mean ± SEM and are representative of two independent experiments (*p ≤ 0.05, **p ≤ 0.01, ***p ≤ 0.001; n = 4).

We next tested whether a NaCl-hypertonic micromilieu affects the upstream cellular mechanisms before DC-T cell interaction, using OVA derivates as model antigens. First, we analyzed OVA uptake and observed a time-dependent increase of endocytosis of fluorescently labeled OVA by BMDCs from hypertonic medium (370 mOsm, 450 mOsm) in comparison to the isotonic, control group (Fig. 2a–d). Of note, apart from a population with moderate OVA uptake, we observed that a subpopulation of the DCs internalized high amounts of OVA, which in previous studies has been demonstrated to be mediated by the mannose receptor (MR)^25^. Consecutively, a strong enhancement of the BODIPY.FL fluorescence (generated upon intracellular hydrolysis of DQ-OVA) in the BMDCs from hypertonic medium was evident, reflecting increased antigen degradation (Fig. 2e). Interestingly, this effect was independent of MR (Fig. 2f), which correlates with previous observations showing that MR-internalized antigens are rescued from lysosomal degradation and that rapid antigen degradation might result from other endocytosis mechanisms^25, 26^. Additionally, differences in antigen degradation were not due to altered overall acidification of endosomes, as the intra-endosomal pH was not influenced by hyperosmolarity (Suppl. Fig. 3a). We further explored the cross-presentation of OVA using the 25D1.16 antibody that specifically recognizes the OVA epitope SIINFEKL when loaded on MHCI. The flow cytometric analysis revealed increased surface expression of the MHCI-SIINFEKL molecules (Fig. 2g, left). SIINFEKL-loaded BMDCs showed a similar tendency (Fig. 2g, right). Concordant to the OVA - uptake and - processing data, these results point to an enhanced cross-presentation by the BMDCs matured in NaCl-hypertonic medium.

### Altered co-signaling receptor expression and immune synapse cytokine secretion by BMDCs exposed to hypertonic stress are not sufficient to hinder the CD8^+^ T cell activation

**Figure 3:**
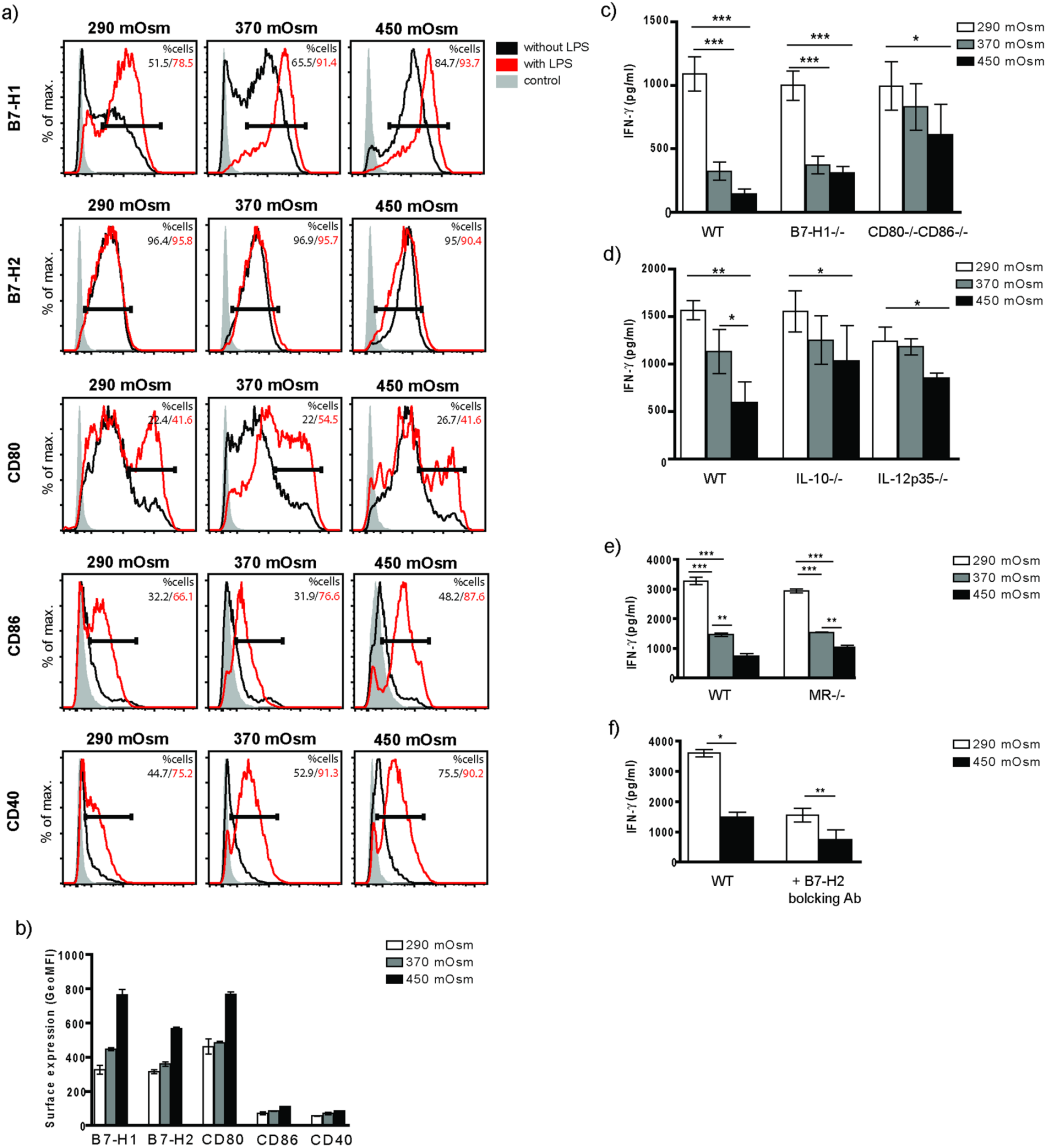
High salt micromilieu modifies surface expression of co-stimulatory and co-inhibitory molecules on BMDCs. (**a**) Representative histogram plots displaying relative surface expression intensity for B7-H1, B7-H2, CD80, CD86 and CD40 (black contour represents BMDCs raised in endotoxin-free conditions; red contour shows receptor expression by BMDCs stimulated by LPS) with (**b**) corresponding statistical graph showing geometric mean fluorescent intensity (GeoMFI) of surface receptor expression without LPS stimulation. Histogram depicts all events after gating on living cells. (**c**–**e**) BMDCs of B7-H1^−/−^, CD80-CD86^−/−^, IL-10^−/−^, IL-12p35^−/−^ and MR^−/−^ origin were raised in indicated NaCl-hypertonic conditions and prepared as described in Fig. 2 for the T cell activation assay. B7-H2 on WT BMDCs was blocked as described earlier (**f**). IFN-γ was measured from cell culture supernatants. Statistical data are expressed as mean ± SEM and are representative of two independent experiments (*p ≤ 0.05, **p ≤ 0.01, ***p ≤ 0.001; n = 6 for B7-H1^−/−^, CD80-CD86^−/−^ and IL-12p35^−/−^; n = 4 for IL-10^−/−^, MR^−/−^ and B7-H2 experiments).

We next tested whether hyperosmotic conditions influence the DC – T cell immune synapse pattern. We could observe enhanced surface expression of the co-inhibitory molecule B7-H1 and conditionally two-directional co-signaling receptors B7-H2 and CD80, with minor changes in CD86 and CD40 expression (Fig. 3a,b). We have previously shown strongly decreased IL-12p70 and increased IL-10 secretion by BMDCs exposed to high salt under LPS stimulation, both potentially influencing immune synapse signaling^14^; now we have performed intracellular stainings for IL-12p70 and IL-10 and could observe the same tendency in endotoxin-free conditions as well (data not shown). Moreover, MIP-2 secretion was significantly upregulated in BMDCs raised in hypertonicity (Suppl. Fig. 3b). To explore whether these co-signaling changes might be responsible for abrogated cross-priming, we used DCs derived from bone marrows of corresponding receptor- or cytokine-deficient mice. None of the blocking approaches could rescue the cross-priming phenotype (Fig. 3c–h). In addition, cross-priming was strongly blocked in BMDCs gained from MR^−/−^ mice as well, showing that potential ‘overload’ of BMDCs from high salt medium due to increased OVA uptake is not likely to be responsible for decreased priming.

### NaCl-hypertonic stimuli induce H-2Kb-SIINFEKL surface cluster formation in BMDCs

**Figure 4:**
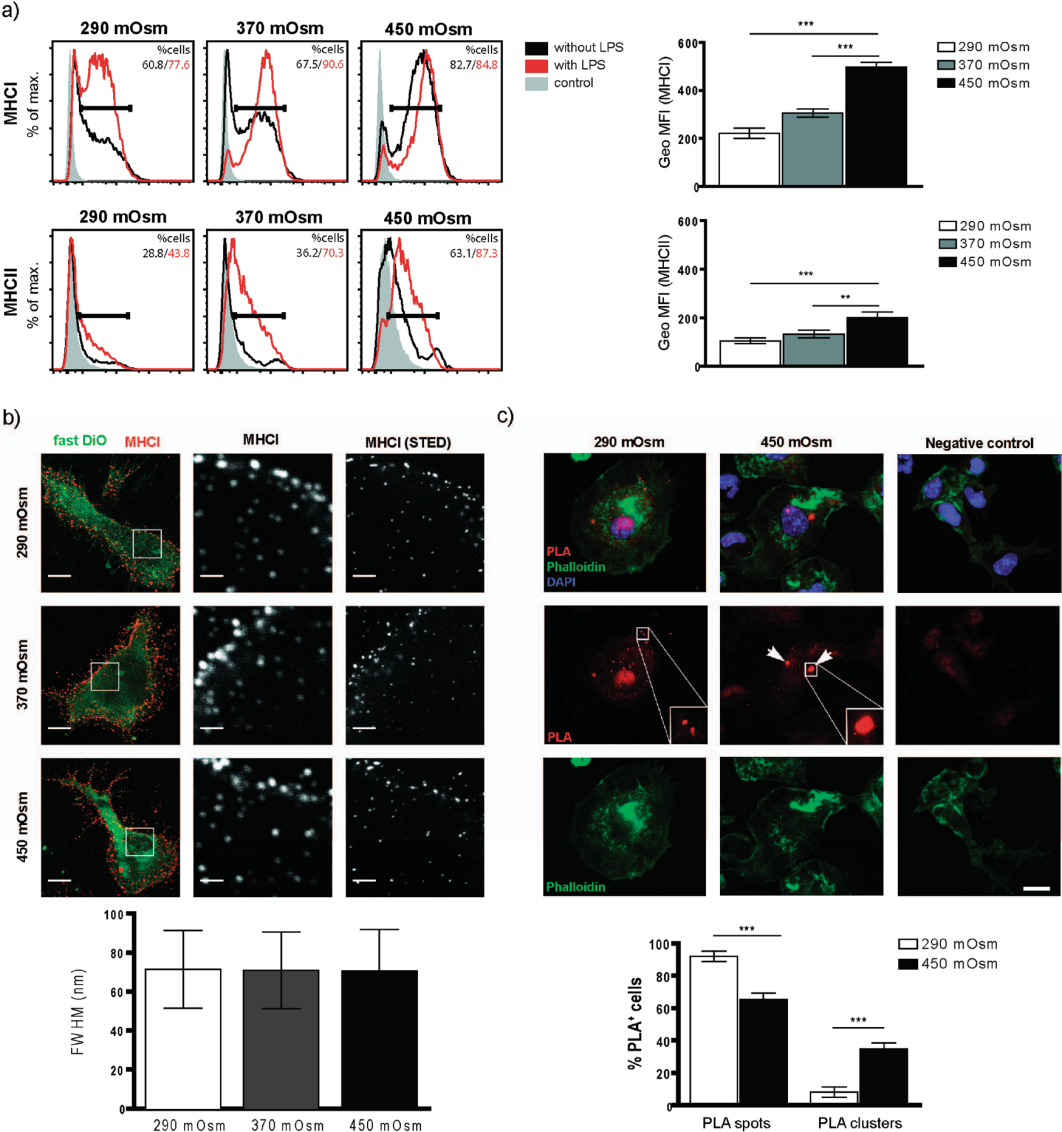
BMDCs matured in NaCl-rich microenvironment have higher surface expression of MHCI and show surface clustering of MHCI-SIINFEKL complex upon OVA uptake. (**a**) Representative histogram plots displaying relative surface expression intensity of MHCI (upper graph) and MHCII (lower graph) by BMDCs from indicated media osmolarities (black contour represents BMDCs raised in LPS-free conditions; red contour shows receptor expression by BMDCs stimulated by LPS) with corresponding statistical graphs showing GeoMFI data for LPS-free samples (right). (**b**) MHCI was immunostained for stimulated emission depletion (STED) imaging in BMDCs cultured in medium with an osmolarity of 290 mOsm, 370 mOsm or 450 mOsm, without addition of OVA. Column 1 shows standard confocal micrographs of MHCI clusters (red) in cells with Fast DiO membrane counterstaining (green) with scale bars indicating 5 μm. In the areas marked with a white box, MHCI was imaged using STED superresolution microscopy (column 3) and for comparision with the same settings without STED (column 2). Scale bars indicate 1 μm (101–171 clusters from 13–15 cells per condition, one experiment). Analysis of the MHCI cluster size revealed a similar full width at half maximum (FWHM) of around 71 nm for all three culturing conditions. (**c**) BMDCs cultured in 290 mOsm (left column) and 450 mOsm (middle column) plated on cover slips were incubated with 0.5 mg/mL OVA for 4 hours, fixed and stained in a proximity ligation assay (PLA) with anti-MHCI antibody and 25D1.16.APC antibody. PLA-spots (visible only in the case of specific binding of both antibodies in each other’s proximity) and –clusters (defined as a convincing grouping of multiple spots) were detected using Keyence BZ-9000 microscope (enlarged in the areas marked with a white box). A weak red fluorescence background in the nuclear area is occasionally observed. Scale bar indicates 10 µm. The right column shows negative control, omitting the anti-MHCI antibody. Statistical analysis (lower graph) demonstrates distribution of spots and clusters in 290 mOsm and 450 mOsm groups, displayed as % of PLA-positive cells having either spots or clusters. The data are displayed as mean ± SEM from two pooled experiments (***p ≤ 0.001, n = 19–30 high power fields).

**Figure 5:**
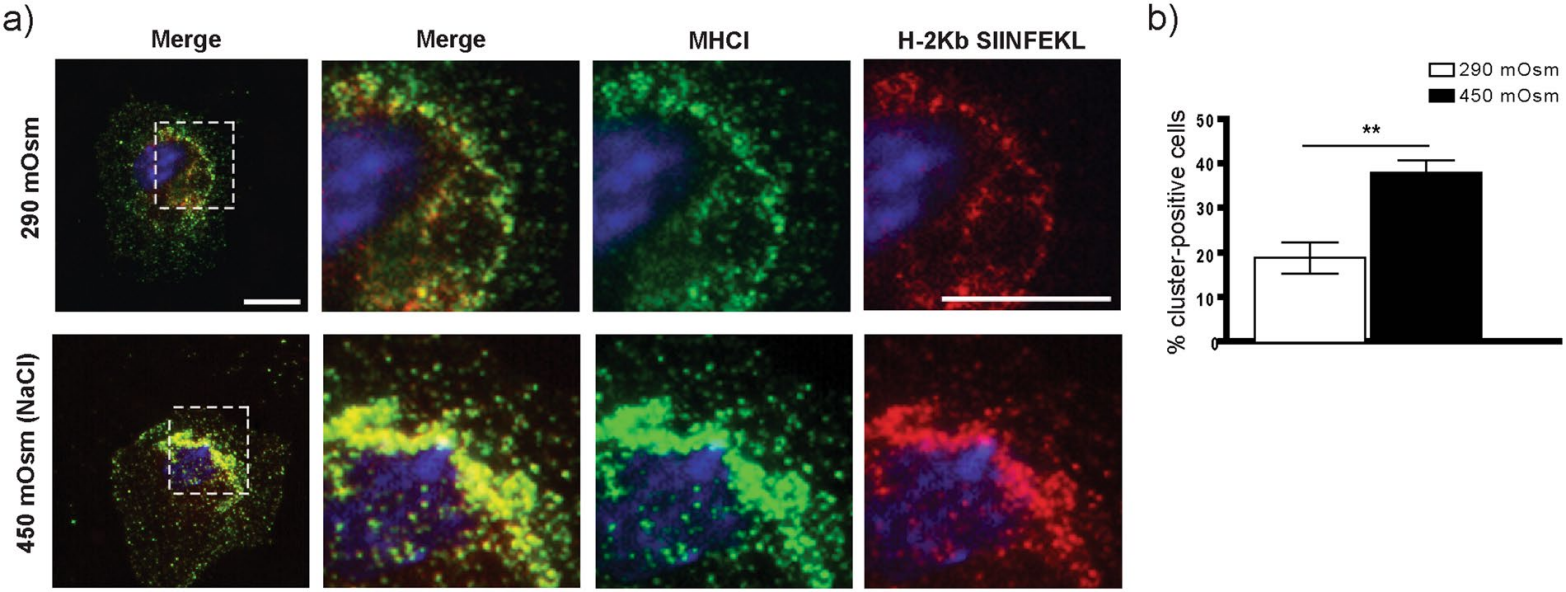
BMDCs raised in hypertonic microenvironment demonstrate clustering of MHCI and SIINFEKL-loaded MHCI molecules. A method alternative to the PLA assay was applied to confirm the receptor-peptide cluster formation upon exposure of BMDCs to high NaCl. (**a**) Immunofluorescent photomicrographs obtained using double surface staining for MHCI (H-2Db with a secondary Alexa Fluor 488-labeled antibody) and for H2-Kb-SIINFEKL complex (25D1.16 antibody directly fluorescent labeled by APEX Alexa Fluor 555 antibody labeling kit). (**b**) The statistical data are presented as mean ± SEM (**p ≤ 0.01, n = 16 high power fields).

We next analyzed whether exposure of BMDCs to hyperosmolar micromilieu affects the surface expression of MHCI. Both MHCI and MHCII (which were transported towards the cell surface upon treatment with LPS as previously reported^27^) exhibited a dosage-dependent upregulation in salt-exposed BMDCs (Fig. 4a). Remarkably, MHC I was mainly upregulated only in a subpopulation of BMDCs. However since hyperosmolarity correlates with increased MHC I expression but with decreased T cell activation, it seems unlikely that differences in MHC I expression alone are responsible for altered T cell priming. To test whether exposure of BMDCs to hypertonic condition alters DC-T cell interaction, we evaluated the time of DC-T cell contact upon pulsing with OVA. We could observe a reduction in DC-T cell interaction time using DCs from hypertonic conditions (Suppl. Fig. 4; Suppl. movie), albeit statistically not significant at the 5% level. As our cross-priming results seemed to contradict the apparently increased expression of MHC molecules, we postulated that increased MHCI generation together with increased processing of endocytosed OVA may lead to formation of dysfunctional MHCI-SIINFEKL complexes in high-salt DCs. Using STED microscopy, we have looked for altered cluster formation of MHCI molecules on the surface of BMDCs developed in isotonic or hypertonic conditions without exposure to (OVA) antigen. We could not observe significant clustering differences between the groups (Fig. 4b). We further explored surface expression of MHCI-antigen complex upon exposure of BMDCs to OVA. For this purpose, we applied a proximity ligation assay (PLA) using two different antibodies - one directed against the SIINFEKL epitope loaded on MHCI and one against MHCI itself. Only if both antibodies bind their target in close proximity to each other, the amplified PLA signal can be detected. This technique has been used to increase staining sensitivity and reduce fluorescent background^28–30^. Using this assay, surface presentation of SIINFEKL in the complex with MHCI molecule can be detected in form of single spots or clusters of spots. Whereas under normosmolar conditions, antigen-loaded MHC I molecules were distributed in a spotted pattern, we could observe the formation of large surface clusters of SIINFEKL-loaded MHC I molecules in high osmolarity-exposed samples (Fig. 4c). We could confirm these results in our second approach, applying standard double immunofluorescent staining using anti-MHCI in combination with a secondary Alexa 488 labeled antibody, and Alexa 555 directly-labeled 25D1.16 antibody (Fig. 5).

### Hyperosmolarity-induced inhibition of cross-priming is mediated by TRIF signaling

**Figure 6:**
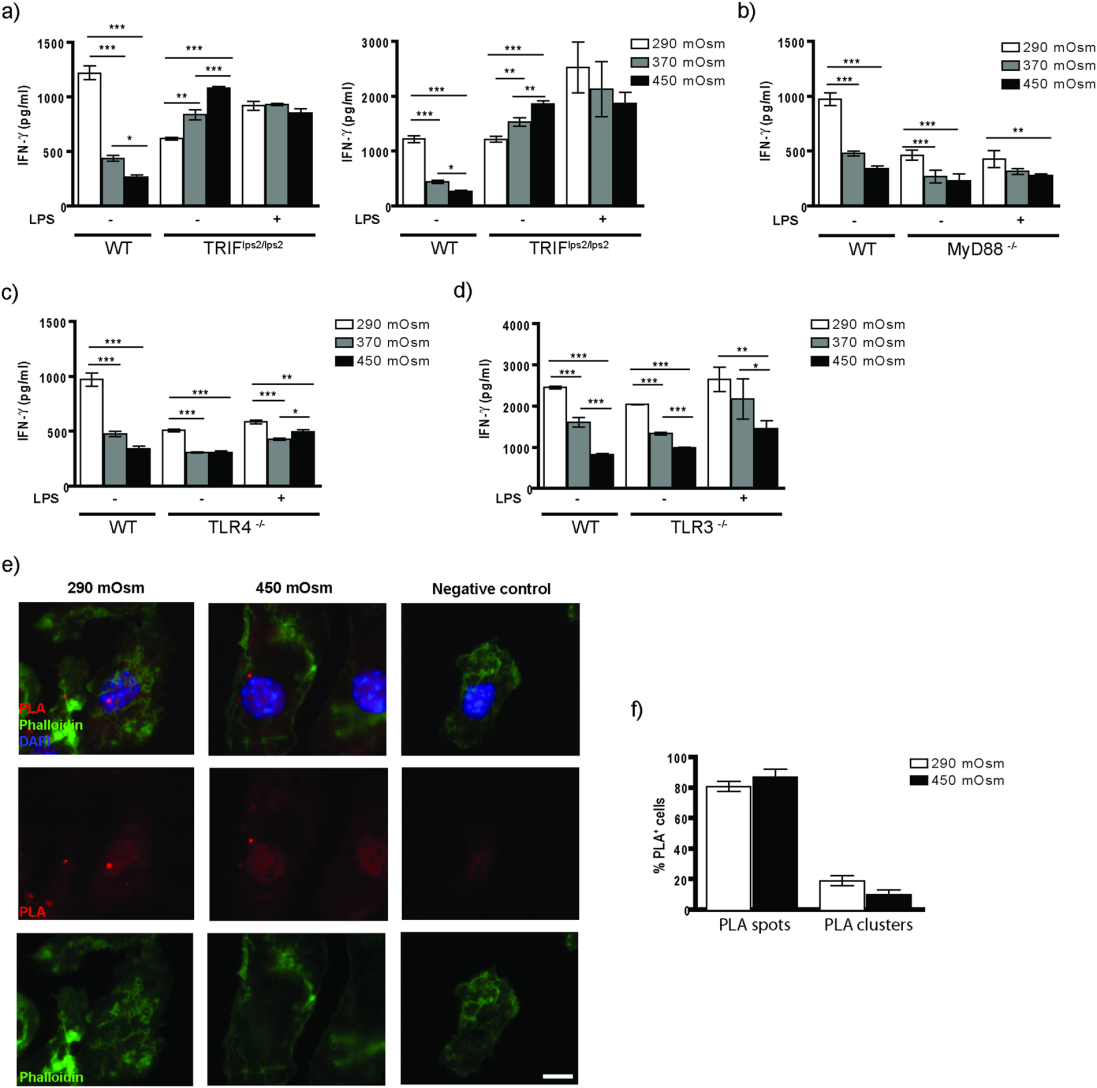
TRIF signaling deficiency abrogates the effect of hypertonicity on CD8^+^ T cell activation. (**a**) BMDCs of TRIF^lps2/lps2^ origin matured in isotonic or indicated NaCl-hypertonic conditions were used in OVA/OTI T cell activation assay as described (**a**, left). Alternatively, BMDCs matured in isotonic medium were stimulated with NaCl solely during OVA uptake (2 hours) and used in the same assay (**a**, right). (**b–d**) Cross-priming of OTI cells by BMDCs raised in different osmolarities, gained from MyD88^−/−^, TLR4^−/−^ and TRIF^−/−^ mice, respectively. Displayed graphs are representative of minimum two independent experiments (*p ≤ 0.05, **p ≤ 0.01, ***p ≤ 0.001; n = 4). (**e**) BMDCs of TRIF^lps2/lps2^ origin matured in 290 mOsm (left column) and 450 mOsm (right column) media were used in PLA-assay as described. Negative control (right column) represents probes with application of only one antibody (25-D1.16.APC antibody was obeyed). The statistical data (**f**) demonstrate % of PLA-spot or –cluster positive cells within total PLA-positive BMDC population (displayed as mean ± SEM; representative of two pooled experiments, ***p ≤ 0.001, n = 21 high power fields).

**Figure 7:**
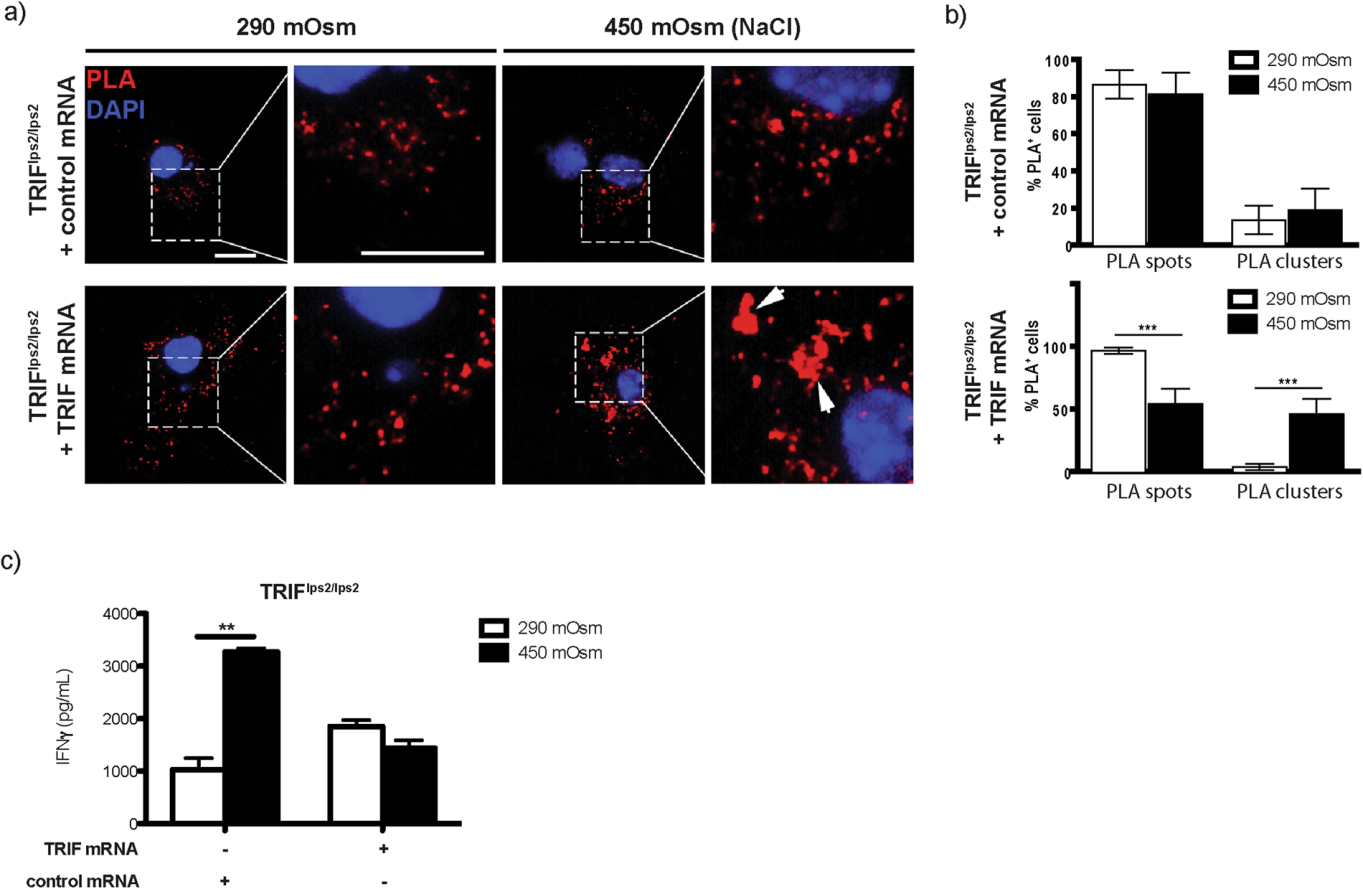
Overexpression of TRIF in TRIF-deficient BMDCs rescues the phenotype leading to both increased surface cluster formation and reduced OTI activation upon exposure to high salt. BMDCs from TRIF^lps2/lps2^ exposed to isotonic or hypertonic (450 mOsm) conditions were electroporated with 15 µg control or TRIF mRNA. Cells were further incubated with OVA grade VII (1 mg/mL) for 4 hours, followed by overnight (17 hours) incubation with OTI cells. (**a**) Spots and clusters on BMDC surface upon exposure to hypertonic medium were assessed by PLA assay. The statistical graphs (**b**) show % of PLA-spot or –cluster positive cells in TRIF-deficient BMDCs overexpressed for TRIF (lower lane) or exposed to control mRNA (upper lane). (**c**) BMDCs obtained from the bone marrows of mice used for PLA assay were in parallel applied in OVA/OTI activation assay (IFN-γ secretion measured from cell culture supernatants, displayed as mean ± SEM; ***p ≤ 0.001 and **p ≤ 0.01, n = 3).

To explore whether TLR-signaling plays a role in high salt-mediated reduction of cross-priming, we used BMDCs from TLR4^−/−^, TLR3^−/−^, MyD88^−/−^ and TRIF^lps2/lps2^. The cross-priming was fostered both in TRIF-deficient BMDCs exposed to high salt during the last four days of differentiation and in cells exposed to NaCl solely during OVA uptake (Fig. 6a). Interestingly, this effect was statistically highly significant in the endotoxin-free group (following the increase in OVA uptake, presentation), whereas no significant difference appeared in the LPS-treated group (Fig. 6a). MyD88 deficiency did not influence the cross-priming results obtained in WT cells (Fig. 6b). To our surprise, experiments with TLR4^−/−^ and TLR3^−/−^ DCs have not given results similar to those from TRIF-impaired cells (Fig. 6c,d). We next tested MHCI-SIINFEKL cluster formation in TRIF-deficient BMDCs exposed to high salt by performing PLA assays; we could not detect differences in cluster formation between the two osmolarity groups (Fig. 6e), implying that TRIF might mediate MHCI-Antigen surface clustering and that cluster formation is potentially directly responsible for an ineffective DC-T cell contact. To further substantiate a role of TRIF in this process, we overexpressed TRIF in TRIF-deficient BMDCs. We could clearly demonstrate that overexpression of TRIF in these cells indeed led to impaired T cell activation, resembling the WT phenotype (Fig. 7c). PLA analysis of BMDCs from the same mice showed a strongly increased cluster formation on the surface of TRIF-overexpressing cells in comparison to control mRNA-treated TRIF-deficient cells, formally proving a decisive role of TRIF within this process (Fig. 7a,b).

### Intact TRIF signaling is important for a cortex-oriented lymphocyte infiltration during renal allograft rejection

In kidney, DCs that populate renal medulla are exposed to increasing concentrations of osmolytes such as sodium. We have previously shown that leukocytes, including DCs, in acute renal allograft rejection in WT mice distribute predominantly in renal cortex and have provided indirect evidence that increasing interstitial osmolarity towards inner medulla might mediate this phenomenon^14^. As renal DCs may present antigens to prime T cells^31^, we applied here murine renal allograft transplantation as an *in vivo* model involving both high osmolarity gradient and infiltration of T lymphocytes. In the light of current *in vitro* results, we investigated whether TRIF or MyD88 deficiency of recipient mice might abrogate the cortically oriented T cell recruitment. As reported^32^, donor BALB/c kidneys transplanted to both TRIF- and MyD88-deficient recipients showed an overall lower degree of inflammation (with less functional improvement in TRIF- than in MyD88-deficient mice) than the kidneys transplanted to WT recipients (Suppl. Fig. 5a,b). In line with our *in vitro* results, in contrast to the BALB/c-C57BL/6 group, no significant difference in the intensity of CD3^+^ cell infiltration between cortical and outer medullary compartment of BALB/c kidneys transplanted to TRIF-deficient (but not MyD88^−/−^) recipients could be observed (Suppl. Fig. 5a,b). These results underline TRIF as a potentially important player in osmolarity-mediated T cell recruitment.

### NaCl-rich micromilieu modulates the cross-presentation-related gene expression signature in bone marrow-derived dendritic cells

We performed transcriptional profiling of WT and TRIF-deficient BMDCs raised in normosmolar 290 mOsmol/L (mOsm) or NaCl-hyperosmolar (450 mOsm) medium (Fig. 6, Suppl. Table 1). Microarray analysis of genes clustered to *Class I MHC Mediated Antigen Processing Presentation Pathway* (Reactome) revealed a predominant downregulation of genes involved in proteasome degradation and coding for ubiquitin-conjugating enzymes upon exposure to high salt medium. On the other side, single genes coding for chains of H-2 class I histocompatibility antigen (members of the MHCI family: *H2*-*K1* and *H2*-*M3*) showed a significant upregulation upon hypertonic NaCl stimuli (Suppl. Fig. 6a). It was evident that, despite a predominant overlap of (450 mOsm vs. 290 mOsm) differentially regulated genes between WT and TRIF-deficient BMDCs, single genes were not differentially regulated in both groups (Suppl. Table 1). Among them, we could observe a prominent NaCl-triggered upregulation of translocon Sec61 subunits (Sec61a1 and Sec61a2) and upregulation of H2-M3 in WT, but not in TRIF BMDCs (Suppl. Fig. 6b, Suppl. Table 1).

## Discussion

Mononuclear phagocytes may adapt towards changes in the micromilieu by acquiring tissue-specific, disease-specific genotypic and functional signatures^33–35^. Biophysical factors, including local osmolarity, may play an important role in shaping the immune response in diverse disease settings^14, 36^. Increasing evidence on the role of hypertonicity on Mϕ activation demonstrates the complexity of interaction between mononuclear phagocytes and microenvironmental osmolytes^14, 21–24^. Classical ‘M1’ stimulation leads to a proinflammatory Mϕ activation with consecutive nitrate production and bactericidal activity; increased Na^+^ concentration has been reported to boost the M1 cascade during infection, thereby facilitating the antimicrobial host defense via p38/MAPK-dependent NFAT5 signaling^23^. Accordingly, a recent study reported that high dietary salt intake promotes pro-inflammatory Mϕ to aggravate CNS autoimmunity^37^. Others have detected NaCl-induced augmentation of proinflammatory Th17 phenotype in an autoimmune disease setup, mediated by SGK1 and NFAT5^24^. In addition, LPS-activated Mϕ have been shown to respond to hyperosmotic stress through mitochondrial ROS-triggered NLRP3 and NLRC4 inflammasome activation, consecutively enhancing IL-1ß secretion and promotion of the Th17 response^22^. On the other hand, ‘M2’-activated Mϕ upon exposure to high salt may exhibit reduction of suppressive capacity typical for an ‘alternative’ signature, albeit without acquiring ‘M1’ phenotype, and independent on NFAT5 signaling^21^. Our previous study has shown NFAT5-independent chemotaxis of Mϕ, but not DCs, towards high salt concentration^20^. In the first study dealing with the role of NaCl-induced hypertonicity on DCs we recently reported that hyperosmotic stress polarizes DCs towards a ‘M2’-like phenotype without losing DC surface receptor markers, suggesting a decreased alloreactivity of renal medullary DCs compared to ‘isotonic’ cortical DCs^14^. Together, these observations support the notion that NaCl does not exhibit a uniform effect on the mononuclear phagocyte system, but rather interacts with other microenvironmental players to trigger tissue- and pathology-related pathways. We provide here evidence for a NFAT5-independent inhibition of the cross-priming capacity of BMDCs upon exposure to high osmolarity of either ionic (NaCl) or nonionic (mannitol) origin, resulting in an abolished antigen-specific T cell response independent of infection-related microenvironmental signals.

We have now shown that hyperosmotic stress may influence multiple steps of the antigen cross-presentation pathway, including endocytosis, processing, presentation, expression of co-signaling receptors/immune synapse-associated cytokines and finally cross-priming. High salt micromilieu can significantly enhance MR-dependent endocytosis of soluble OVA with consecutively increased OVA processing and presentation; nevertheless a strong inhibition of the cross-priming capacity was evident in MR^−/−^ mice as well, suggesting that possible ‘overload’ of DCs with an antigen is not likely to play a major role in the hindering effect of NaCl on T cell activation and confirms the observation of MR-independent cross-presentation pathways^38^. The efficiency of cross-presentation has also been shown to be influenced by the acidification state of the antigen-containing endosomes, in which a rapid acidification results in strong activation of lysosomal proteases and the quick removal of epitopes putatively intended for cross-presentation^2, 39^. Here, we have demonstrated that hyperosmolarity does not influence overall acidification of endosomes, meaning that differences in cross-presentation must be due to other mechanisms.

Moreover, despite upregulation of several co-inhibitory signals on the DC side of the immune synapse, depletion of single ones could not revert the effect of hyperosmolar stress on cross-priming, not necessarily excluding a combined effect. On the other hand, we have provided evidence that blockade of TRIF signaling, but not MyD88-deficiency, reverses the effect of high salt stress on DCs compared to the wildtype situation in both endotoxin-free and LPS-pulsed cells. Remarkably, the effect of TRIF signaling seemed to be even higher in the absence of LPS and under these conditions, hyperosmolarity seemed to induce even an increase in T cell priming. Since under isotonic conditions, TRIF signaling is responsible for efficient translocation of Sec61 towards antigen-containing endosomes and hence for efficient cross-presentation^10^, under hyperosmolarity, different mechanisms might be responsible to enable such intracellular Sec61 translocation. Alternatively, high osmotic conditions might also influence the cross-presentation pathway per se. Under normal osmotic conditions, OVA is mainly cross-presented by BMDCs via the endosome-to-cytosol pathway^3, 40^, which needs the presence of Sec61 in endosomes^10^. However, it is conceivable that hyperosmolarity enables the vacuolar cross-presentation pathway, in which antigens are not translocated into the cytosol but rather degraded within endosomes and loaded onto MHC I molecules there^2, 41–43^, and therefore enables Sec61-independent cross-presentation. Future experiments focusing on the exact molecular mechanisms regulating Sec61 translocation will have to shed light on the underlying molecular pathway.

Remarkably, neither TLR4- nor TLR3-deficient DCs mirrored the effect of TRIF-deficiency, suggesting that the mechanism(s) leading to TRIF-mediated inhibition of cross-priming are mediated via a to-date unknown pathway. We have previously shown that an efficient cross-presentation requires intact TRIF signaling^3^. Importantly, our *in vivo* data using an allotransplantation approach has shown a similar distribution of T lymphocytes across isotonic and hypertonic kidney compartments upon transplantation to TRIF-deficient recipients, supporting the notion that also *in vivo* TRIF mediates adaptive response of DCs to hypertonic conditions. Moreover, our data on external antigen-dependent MHCI-SIINFEKL membrane surface clustering upon DC exposure to hypertonic stress implicate that cluster formation might play a role in inhibition of CD8^+^ T cell activation. One possibility is that this phenomenon leads to an aberrant DC-T cell contact. Our attempts to address this point via measuring the duration of DC-T cell contact in an *in vitro* flow system showed a decreasing tendency towards high osmolarity-matured DCs. In addition, MHCI-antigen cluster formation was not evident in TRIF-deficient DCs, suggesting a link between receptor-antigen clustering and cross-priming effect of high salt. Further research will be necessary to elucidate hyperosmolarity-induced signaling steps upstream of TRIF as well as molecular links between TRIF signaling and MHCI-antigen cluster formation.

A number of pathologic conditions have been associated with local or systemic hyperosmotic stress, including solid tumors^16, 44^. In our main *in vitro* approach, the high osmolarity micromilieu during BMDC differentiation intended to mimic infiltration of immature monocytes to a potentially hyperosmotic site of inflammation and/or neoplasia, where DC maturation and contact with antigen might take place. Cross-priming by such antigen-loaded DCs may occur following two potential scenarios: T cells can be primed in tertiary lymphoid follicles at the site of lesion – as a response to chronic inflammation or cancer–^45^, or classically upon migration of antigen-loaded DCs to secondary lymphoid organs (lymph nodes, spleen, liver,)^46, 47^. Our alternative approach – exposure of BMDCs to hyperosmotic stress during OVA uptake, or during both OVA uptake and T cell priming – intended to reflect an *in vivo* scenario where antigen-pulsed dendritic cells matured in isotonic micromilieu face hyperosmolar conditions upon migration towards hyperosmolar lymphatic tissue^15^. We report here using both approaches that cross-priming capacity of DCs strongly suffers under hyperosmolar stimuli. This effect does not seem to be NaCl-specific, as biologically inert nonionic osmolyte mannitol, although to a lesser extent, could trigger a significant reduction of T cell activation at 450 mOsm conditions.

The hyperosmotic milieu is not necessarily linked to pathology. Physiological osmolarity of the blood (285–295 mOsm) is kept in a tight range via robust regulatory mechanisms^15, 22^. Reports that included direct measurement of tissue osmolarity have demonstrated physiological hyperosmolarity of lymphoid tissue compartments, reaching 330 mOsm-340 mOsm^15^. In the kidney, the medullary compartment is exposed to a physiological osmolarity gradient reaching values of >1000 mOsm (inner medulla)^13^. Furthermore, the homeostatic hyperosmolarity of intervertebral disc and articular cartilage tissue has been reported to vary between 430 mOsm and 496 mOsm^17, 19^ and appears to be crucial for an elevated proteoglycan synthesis^48^. In pathologic conditions affecting kidney or intervertebral disc tissue (renal transplantation, intervertebral disc herniation), inflammatory infiltrate consisted mainly of immature monocytes/Mϕ, with rather depressed lymphocytic- and dendritic cell-pools^14, 18^. Our study contributes to elucidation of the cellular mechanisms involved in immune responses to hypertonic stimuli by demonstrating a NFAT5-independent reduction of the cross-priming capacity of DCs exposed to osmolarity ranges that reflect reported *in vivo* values without an influence ont DC survival. Our findings give insight into a novel mechanism affecting both innate and adaptive immune response within hyperosmolar microenvironments occurring in homeostatic tissue and in tumors: a hypertonicity-triggered suppression of antigen-specific DC-mediated immune response. We propose thus that targeting local hyperosmolarity may support cytotoxic T cell response, potentially contributing to an enhanced anti-tumor immunity.

## Methods

### Mice

C57BL/6 wild type (WT), MyD88^−/−^, TRIF- deficient (TRIF^lps2/lps2^), TLR4^−/−^, NFAT5^flox/flox^CD11c^Cre^, B7-H1^−/−^, CD80/86^−/−^, IL-10^−/−^, IL-12p35^−/−^, MR^−/−^, OTI and OTII mice (8–12 weeks old) were obtained from breeding colonies of the animal facility of the German Cancer Research Center or the University of Bonn. NFAT5^−/−^ and NFAT5^flox/flox^ mice were obtained from Dr. Wolfgang Neuhofer. TLR3^−/−^ mice were purchased from The Jackson Laboratory (Bar Harbor, ME, USA). BALB/c mice were purchased from Charles River (Wilmington, MA, USA). All mice were kept in specific pathogen-free conditions. All mice experiments are carried out in accordance with German laws and regulations concerning work with animals and genetically modified organisms. All experimental protocols for animal experiments were approved and authorized by local Government authorities (Regierungspräsidium Karlsruhe, Baden-Wuerttemberg, Germany).

### Antibodies and reagents

Cytokine secretion was measured from supernatants using mouse CBA Flex-Set assay from BD Biosciences (Heidelberg, Germany) or, in the case of MIP-2, ELISA kit (RayBiotech, Norcross, USA). LEAF™ Purified anti-mouse B7-H2 antibody for *in vitro* B7-H2 blocking was bought from BioLegend (Fell, Germany). Ovalbumin grade VII (OVA) and sodium chloride (NaCl; cell culture tested) were purchased from Sigma-Aldrich (Taufkirchen, Germany). Endotoxin-free ovalbumin (ET-free OVA) was bought from Hyglos (Bernried am Starnberger See, Germany). DQ^TM^ ovalbumin (DQ-OVA) and ovalbumin Alexa Fluor® 488 conjugate (OVA.Alexa488) were purchased from Thermo Fisher Scientific (Waltham, MA, USA). OVA 257–264 (SIINFEKL) peptide was obtained from InvivoGen (San Diego, CA, USA).

### Generation of murine bone marrow-derived dendritic cells challenged with different osmolarities and sorting of ***ex vivo*** splenic dendritic cells

BMDCs were generated using GM-CSF-enriched RPMI medium, as described^49, 50^. On day 3, the medium was supplemented with different volumes of cell culture tested 4 M NaCl solution, to reach the final osmolarity of 340 mOsm, 370 mOsm and 450 mOsm. In parallel, part of the cells was kept in isotonic conditions (290 mOsm). The cells were split on day 4 and maintained in different osmolarities until day 7, when mature BMDCs were subjected to further experiments, as described. Flow cytometric analysis of BMDCs for surface receptor expression was performed using BD FACSCalibur^TM^ and BD FACSLSRII^TM^, using FlowJo 7.5.3 software. Dendritic cells from spleens of C57Bl/6 mice were purified using CD11c MicroBeads UltraPure and a magnetic separation system, according to manufacturer’s protocol (Miltenyi Biotec, Bergisch Gladbach, Germany).

### Gene expression profiling

Gene expression profiling from BMDCs was performed using GeneChip Mouse Gene 1.0 ST Arrays (Affymetrix, Santa Clara, CA, USA). RNA was isolated using the RNeasy Micro Kit (Qiagen, Hilden, Germany), including on-column DNAse digest. Quantitative and qualitative analysis of the isolated RNA was done on an Agilent 2100 Bioanalyzer (Agilent, Santa Clara, CA, USA). Equal amounts of cDNA were fragmented and labeled using the Nugen Encore Biotin Module (Nugen Technologies Inc.) and hybridized on the aforementioned arrays.

### Ovalbumin uptake, degradation and presentation assays

For uptake experiment, BMDCs from WT or MR^−/−^ mice matured in 290 mOsm, 370 mOsm or 450 mOsm media were exposed to fluorescent OVA conjugate (10 µg/mL for 5 min, 30 min or 120 min). OVA degradation was assessed using DQ-OVA (10 µg/mL for 4 hours) and quantified by measuring intensity of BODIPY.FL fluorescence (raised upon proteolytic degradation of OVA). For antigen presentation experiments, OVA (2 mg/mL) or SIINFEKL (1 µg/mL) were incubated with BMDCs for 17 hours (overnight) and surface presentation of SIINFEKL/H-2Kb complex was evaluated using APC-conjugated 25D1.16 antibody (eBioscience, Frankfurt, Germany).

### T cell activation assays

*In vitro* T cell activation experiments were performed as described^36^. Briefly, BMDCs from WT or knockout mice matured in isotonic or NaCl-hypertonic media were incubated with 1 mg/mL OVA (alternatively ET-free OVA) with LPS (10 ng/mL) or vehicle (PBS) for 4 hours and subsequently co-cultured with B3Z, OTI, or OTII T cells^3^. In one experimental setting, BMDCs from WT mice were pulsed with blocking anti-B7-H2 antibody (10 µg/mL for 17 hours)^51^. T cells activated by SIINFEKL-loaded (1 µg/mL) BMDCs were used as positive control. Alternative approaches included: *ex vivo* splenic magnetically sorted DCs instead of BMDCs; mannitol as a hyperosmotic reagent during BMDC differentiation (days 3–7) instead of NaCl; pulsing BMDCs raised in isotonic medium with NaCl solely during OVA uptake; or during both uptake and T cell priming. To quantify T cell activation, supernatants were removed after 17 hours of co-culture and assayed by BD CBA Flex-Set for IL-2 (B3Z) or IFN-γ (OTI and OTII) using a FACSCalibur. To directly evaluate T cell proliferation, OTI and OTII T cells were labeled for 15 min with 1 μM CFSE, thoroughly washed, and co-cultured with BMDCs for 96 hours. Proliferation was determined by flow cytometric analysis of CFSE dilution^52^.

### Overexpression of TRIF

BMDCs were transfected with mRNA as described previously^3^. Briefly, mRNA was generated using the T3 mMESSAGE mMACHINE kit and the polyA tailing kit (both Life Technologies). 200,000 BMDCs were electroporated with 15 μg mRNA encoding TRIF or GFP as control with a square wave pulse of 300 V and 6 m sec. T cell activation assays or PLA experiments were performed 1 h after electroporation.

### Analysis of intra-endosomal pH

BMDCs were incubated with 1 μM LysoSensor Green DND 189 (Thermo Fischer Scientific) for 1 h before fluorescence intensities were determined by flow cytometry.

### Membrane surface detection of MHCI clusters by stimulated emission depletion (STED) imaging

Cells were cultered in BMDC media of different osmolarity (290, 370, 450 mOsm) on high precision coverslips (Marienfeld, Lauda-Königshofen, Germany) for 16 hours. Subsequently, cells were rinsed once with PBS, fixed with PBS + 1% PFA fixed (RT 15 min), quenched with PBS + 50 mM ammonium chloride for 20 min at RT, washed 3x with PBS and blocked with PBS + 5% BSA for 1 hour at RT. Staining and embedding was performed as described previously using a mouse anti-mouse MHCI-PE primary antibody (clone 28-14-8, eBioscience, Frankfurt, Germany) diluted 1:200 in PBS + 5% BSA overnight at 4 °C, a goat anti-mouse Atto647N secondary antibody (#50185, Sigma-Aldrich, Taufkirchen, Germany) diluted 1:200 in PBS + 3% BSA for 3 hours at RT and 0.5 μM Fast DiO (Thermo Fisher Scientific) for membrane counterstaining. STED imaging was performed on a four-channel easy3D STED microscope (Abberior Instruments, Göttingen, Germany) at the STED facility of the Life and Medical Sciences Institute (Bonn) as described previously^53^. For Fast DiO, a pulsed 488 nm laser with 500–520 nm and 532–558 nm detection filtersets was used, for Atto647N, a pulsed 640 nm laser and a 650–720 nm filterset. STED images were recorded utilizing a pulsed 775 nm depletion laser and a pixel size of 15 × 15 nm. MHCI cluster size was assessed in STED images by fitting one-dimensional Gaussians to linescans (1 pixel linewidth) through the centres of selected spots. The full width at half maximum (FWHM) is given as an average of 101–171 clusters from 13–15 cells per condition (one experiment).

### Evaluation of H-2Kb-SIINFEKL surface cluster formation by proximity ligation assay and immunofluorescence staining

PLA was performed according to the manufacturer’s guidelines (Duolink Orange Detection System, Sigma-Aldrich, Taufkirchen, Germany), as described^54^. Briefly, 0.1 × 10^6^ BMDCs were seeded onto coverslips in 24-well plates and incubated at standard cell culture conditions with addition of OVA (1 mg/mL) for 6 hours. The cells were washed with PBS and fixed in 4% PFA for 15 min, followed by blocking with 5% skim milk/PBS. PLA was performed with primary antibodies against MHCI (H-2Db, clone 28-14-8) and H-2Kb-SIINFEKL complex (APC-conjugated, 25D1.16, eBioscience, Frankfurt, Germany); the H-2Db antibody was directly labeled by PLA Probemaker (Sigma-Aldrich, Taufkirchen, Germany). Immunofluorescent staining was performed using H-2Db (clone 28-14-8) in combination with a secondary Alexa Fluor 488-labeled antibody, and 25D1.16 antibody directly fluorescent labeled by APEX Alexa Fluor 555 antibody labeling kit (Life Technologies). Formation of PLA spots/clusters and immunofluorescent images were analyzed by fluorescence microscopy (Keyence BZ-9000 Biorevo, Neu-Isenburg, Germany).

### Immunohistochemistry

Immunohistochemical staining was performed on 3 μm sections of formalin-fixed paraffin-embedded tissue, using anti-mouse CD3-ε goat polyclonal antibody (Santa Cruz Biotechnology, Heidelberg, Germany).

### Statistical analysis

Statistical analysis was carried out using GraphPad Prism 5 (GraphPad Software, Inc., La Jolla, CA, USA). Student’s t test was applied in experiments with two groups of data. Repeated measures one-way ANOVA in combination with Tukey’s multiple comparison test were applied in assays with three or more groups of data. Results were expressed as mean ± SEM. A p value ≤ 0.05 was considered statistically significant.

## Electronic supplementary material

Supplementary material

Supplementary movie
