# Supplementary material for: Hyperosmolarity impedes the cross-priming competence of dendritic cells in a TRIF-dependent manner
Source: Sci Rep. 2017 Mar 22;7:311. doi: 10.1038/s41598-017-00434-y (PMC5428499; doi:10.1038/s41598-017-00434-y)
Supplement: Supplementary file 1 — Supplementary material [file 41598_2017_434_MOESM1_ESM.pdf]

Hyperosmolarity impedes the cross-priming competence of dendritic cells in a TRIF-dependent manner

Zoran V. Popovic[*¶], Maria Embgenbroich[#], Federica Chessa[*], Viola Nordström[*], Mahnaz Bonrouhi[*], Thomas Hielscher[§], Norbert Gretz[±], Shijun Wang[*], Daniel Mathow[*], Thomas Quast[**], Jan-Gero Schloetel[***], Waldemar Kolanus[**], Sven Burgdorf[#1] and Hermann-Josef Gröne[*1]

Running headline: High salt hinders cross-priming

[*] Department of Cellular and Molecular Pathology and [§] Department of Biostatistics, German Cancer Research Center, Heidelberg, Germany; [¶] Institute of Pathology and [±] Medical Research Center, University Hospital Mannheim, University of Heidelberg, Mannheim, Germany; [#] Department of Cellular Immunology, [**] Department of Molecular Immunology and Cell Biology and [***] Department of Membrane Biochemistry, LIMES Institute, University of Bonn, Bonn, Germany.

[1] Co-senior authors

SUPPLEMENTARY INFORMATION

SUPPLEMENTARY FIGURE LEGENDS

Supplementary figure 1. The effect of hypertonic micromilieu on $CD8^+$ T cell activation can be observed independent of the concentration of antigen or LPS stimuli and is not regulated by NFAT5. **a)** BMDCs raised in isotonic or hypersomotic conditions (290 mOsm, 370 mOsm and 450 mOsm) were incubated with OVA grade VII (0.2, 0.5, 1 mg/mL) without or with LPS (10 ng/mL) for 4 hours followed by overnight (17 hours) co-incubation with OTI cells. **b)** BMDCs exposed to different osmolarities were incubated with ET-free OVA (1 mg/mL; left graph) or OVA grade VII (1 mg/mL; right graph) without or with different LPS concentrations (10 ng/mL, 100 ng/mL, 200 ng/mL) for 4 hours, followed by overnight co-incubation with OTI cells (n=3). **c)** BMDCs were developed in isotonic or mannitol-enriched (450 mOsm) medium and applied in an OVA/OTI-activation assay, as described (n=4). **d)** BMDCs derived from bone marrows of NFAT5 $^{-/-}$ mice were developed in isotonic or NaCl-hypertonic conditions and used in the cross-priming OVA /OTI assay as previously described (n=4). Statistical data are expressed as mean ± SEM (*p<0.05, **p<0.01 and ***p<0.001).

Supplementary figure 2. Hyperosmolarity in a pathophysiological range (370 – 450 mOsm) does not influence BMDC viability. BMDCs developed in a moderately hyperosmolar (340 mOsm) medium show reduced cross-priming capacity as well. a) Electron photomicrographs of BMDCs matured in 290 mOsm – 450 mOsm conditions, entotoxin-free (upper lane) or upon LPS stimulation (lower lane). No morphologic hallmarks of cell death can be observed. The cells represent 20 randomly selected areas per condition (scale bars = 2500 nm). **b)** BMDCs from C57BL/6 mice were prepared for the cross-presentation assay as indicated in Fig. 2. Statistical graph showing IFN-γ secretion from OTI

cells cross-primed by BMDCs raised in isotonic, moderately NaCl-hypertonic (340 mOsm) and high NaCl (450 mOsm) medium, with or without LPS (100 ng/mL) stimulation. Statistical data are expressed as mean ± SEM and are representative of minimum two independent experiments (*p≤0.05, **p≤0.01, ***p≤0.001; n=4).

Supplementary figure 3. Analysis of intraendosomal pH in BMDCs matured in hypertonic microenvironment and analysis of MIP-2 secretion by BMDCs from different osmolarities. a) Histogram plots (left) and statistical graphs (right) showing LysoSenso green DND-189 mean fluorescence intensity by BMDCs of WT C57BL/6 origin, developed in NaCl hypertonic conditions as indicated. Statistical data are expressed as mean ± SEM (n=3). **b)** BMDCs matured in isotonic and hypertonic medium were plated at $0.1x10^6$/well in 96-well plates for 6 hours in 100 µl of medium without (290 mOsm) or with corresponding NaCl concentration (to reach final 370 mOsm or 450 mOsm), without or with (100 ng/mL) LPS. After 24 hours of incubation at standard cell culture conditions, the supernatants were collected and analyzed for MIP-2 by ELISA. Data are presented as mean ± SEM (**p≤0.01, ***p≤0.001; n=4).

Supplementary figure 4 . Assessment of duration of DC-T cell contact upon exposure of BMDCs to hypertonic conditions. CFSE-labeled OTI T cells were cultured with FarRed-labeled OVA-loaded wildtype BMDCs generated with different osmolarities. The interaction time between T cells and DCs was monitored by time-lapse microscopy (Supplementary movie – exemplary movie documentation) for around 15 randomly chosen cells. Statistical data are expressed as mean ± SEM and are representative of two independent experiments (n=21).

Supplementary figure 5. TRIF mediates cortical vs. medullary (osmolarity gradient) orientation of infiltrating T lymphocytes during acute renal allograft rejection.
a) Photomicrographs of murine zinc-fixed kidney allograft tissue undergoing acute rejection (day 7 post-transplantation) immunostained for CD3 molecule. (Magnification x400, scale bar indicates 20 µM). **b)** Distribution of $CD3^{+}$ cells in cortex and medulla of donor kidneys (BALB/c) 7 days post-allotransplantation to WT, $TRIF^{lps2/lps2}$ or $MyD88^{-/-}$ mice (in C57BL76 background). CD3-positive cells were counted from three high power fields (x40 objective) per kidney from both cortex and outer medulla (***p≤0.001, **p≤0.01, n=5 mice).

Supplementary figure 6. Transcriptional profiling of BMDCs from WT and TRIF $^{lps2/lps2}$ mice developed under isotonic and hypertonic conditions. Heat map demonstrates differential gene expression of members of *MHC class I-mediated antigen processing and presentation pathway* (REACTOME) upon exposure to hyperosmolar microenvironment, from WT **(a)** or TRIF $^{lps2/lps2}$ **(b)** mice. 290 or 450 = BMDCs differentiated in 290 mOsm or 450 mOsm medium, respectively (n=4).

Supplementary table 1: Genes differentially regulated in BMDCs matured in 450 mOsmol/L vs. 290 mOsmol/L medium (cutoff of genes involved in 'Class I MHC Mediated Antigen Processing and Presentation'). Positive logFC values indicate upregulation (red background) and negative values show downregulation (blue background) of gene expression in BMDCs raised in hyperosmolar medium (450 mOsmol/L); **a)** from WT mice (cutoff of significantly altered genes with p value ≤ 0.05; n=4). **b)** Genes differentially regulated in BMDCs from isotonic and hypertonic medium, with no overlap between WT and TRIF-deficient groups (n=4).

Supplementary movie – exemplary movie documentation: Interaction between OTI cells and OVA-loaded BMDCs. The interaction time between OTI cells (CFSE-labeled; green) and BMDCs (FarRed-labeled; red) was monitored by time-lapse microscopy for around 15 randomly chosen cells in both normosmolar and hyperosmolar (450 mOsm) group. Statistical data (Supplementary figure 3) are expressed as mean ± SEM and are representative of two independent experiments (n=21).

SUPPLEMENTARY EXPERIMENTAL PROCEDURES

Transmission electron microscopy. BMDCs developed in 290 mOsm, 370 mOsm and 450 mOsm conditions were cultured on sterile glas cover slips for 17 hours, incubated with 1 mg/mL OVA for 2 hours and washed by PBS. Transmission electron microscopy was performed as described [1].

Analysis of DC / T cell interaction time by time-lapse microscopy. FarRed-labeled BMDCs and CFSE-labeled OTI cells were mixed at a ratio of 1:4 and subsequently were placed in plastic channel slides (µ-slide; ibidi, Planegg / Martinsried, Germany) coated with 5.0 µg/mL ICAM-1/FC (R&D Systems, Wiesbaden, Germany) following incubation with 12.0 µg/mL goat anti-human IgG antibody (Dianova). Analysis of DC / T cell interaction time was carried out as described previously by the use of time-lapse video microscopy [2].

Renal transplantation. Transplantation of BALBc donor kidneys to WT, $TRIF^{tlr2/tlr2}$ and $MyD88^{-/-}$ mice (C57BL/6 background) was performed as described[3, 4]. Mice were sacrificed on day 7 post-transplantation (n=5/group). Analyses of different parameters from the same sections were previously published[3], without interfering with our topic here.

Bioinformatic analyses. Preprocessing and analysis of gene expression values including pathway analysis was performed as described previously [5, 6].

SUPPLEMENTARY REFERENCES

1. Mathow, D. *et al.* Zeb1 affects epithelial cell adhesion by diverting glycosphingolipid metabolism. *EMBO reports* 16, 321-331 (2015).
2. Semmling, V. *et al.* Alternative cross-priming through CCL17-CCR4-mediated attraction of CTLs toward NKT cell-licensed DCs. *Nature immunology* 11, 313-320 (2010).
3. Wang, S. *et al.* Recipient Toll-like receptors contribute to chronic graft dysfunction by both MyD88- and TRIF-dependent signaling. *Disease models & mechanisms* 3, 92-103 (2010).
4. Zhang, Z. *et al.* Improved techniques for kidney transplantation in mice. *Microsurgery* 16, 103-109 (1995).
5. Chessa, F., Hielscher, T., Mathow, D., Grone, H.J. & Popovic, Z.V. Transcriptional profiling of dendritic cells matured in different osmolarities. *Genomics data* 7, 64-66 (2015).
6. Chessa, F. *et al.* The renal microenvironment modifies dendritic cell phenotype. *Kidney international* (2015).

Supplementary Figure 1.

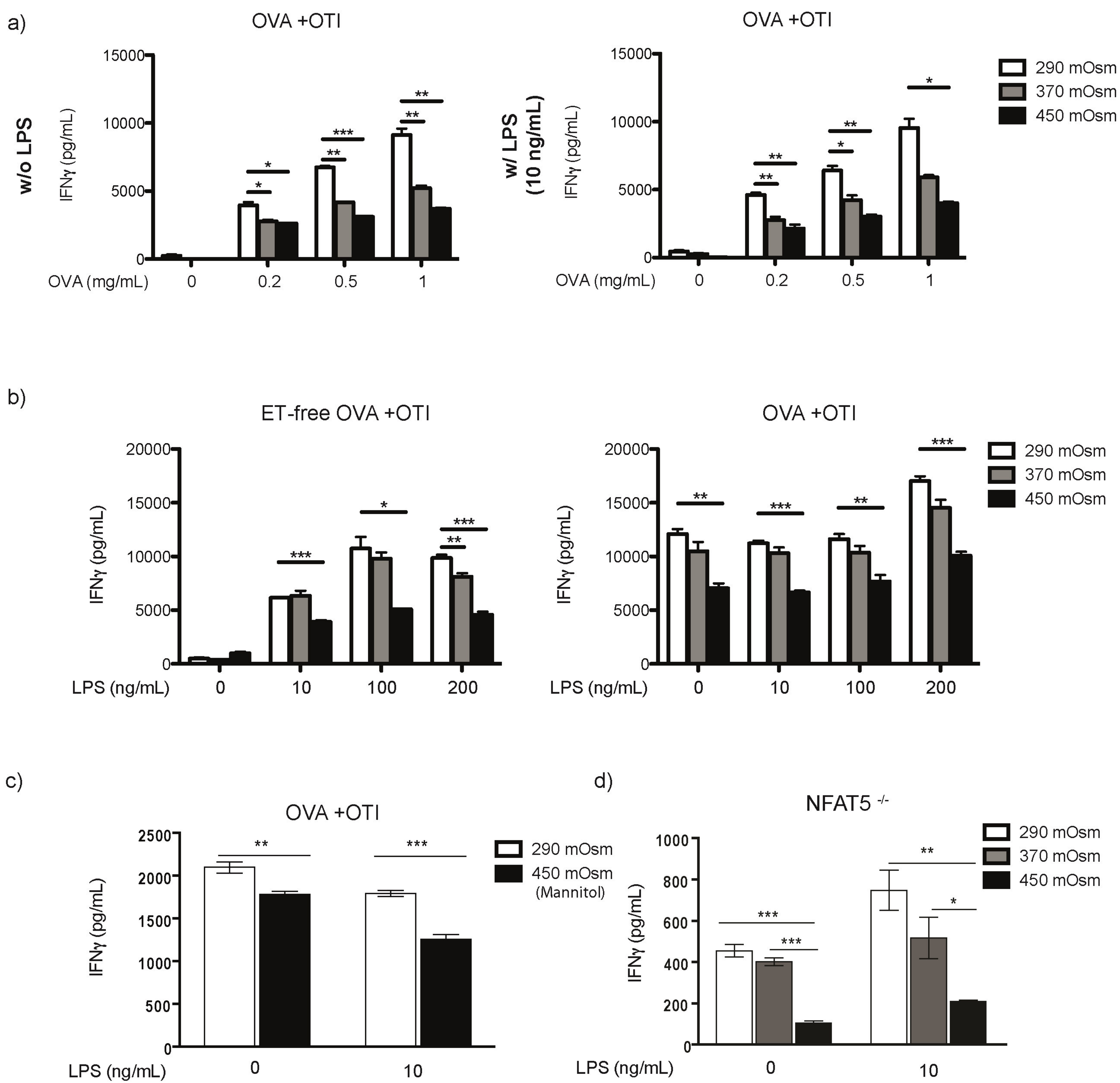

Supplementary figure 2.

a)

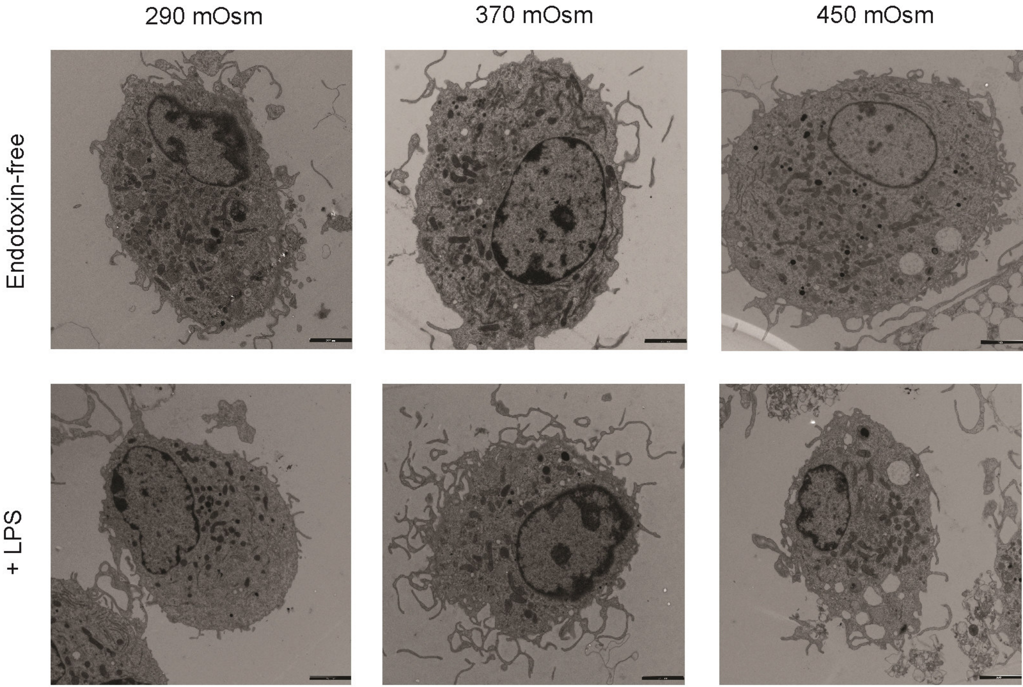

b)

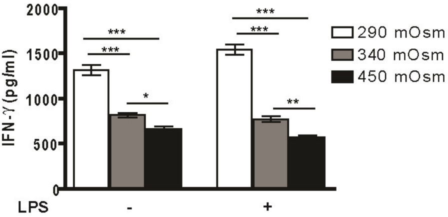

Supplementary figure 3.

a)

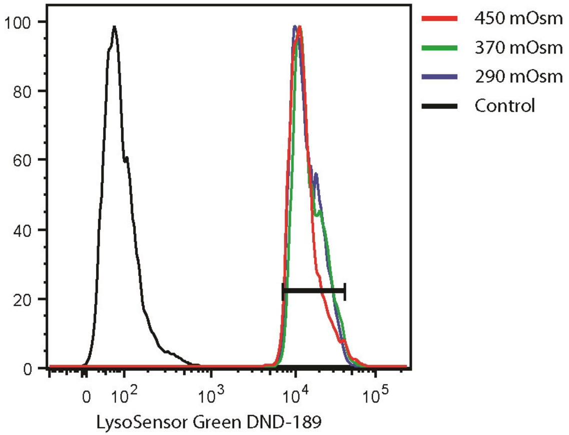

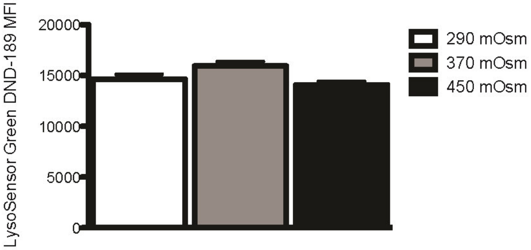

b)

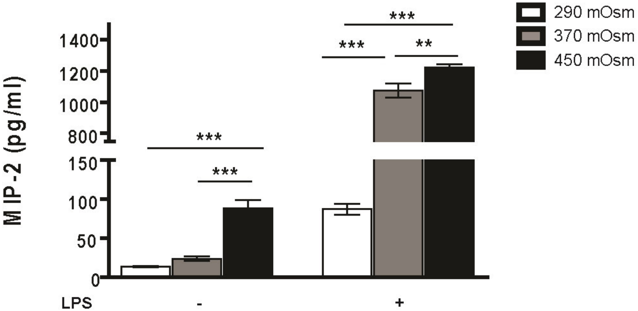

Supplementary figure 4.

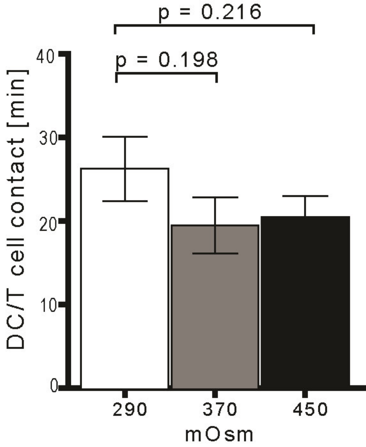

Supplementary figure 5.

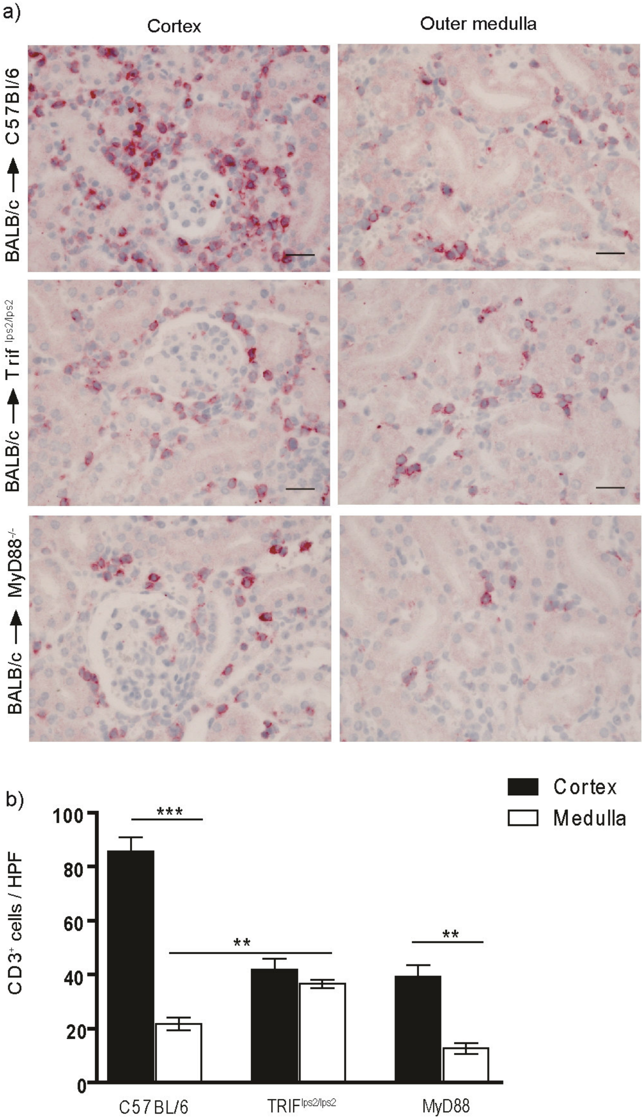

Supplementary figure 6a.

CLASS_I_MHC_MEDIATED_ANTIGEN_PROCESSING_PRESENTATION

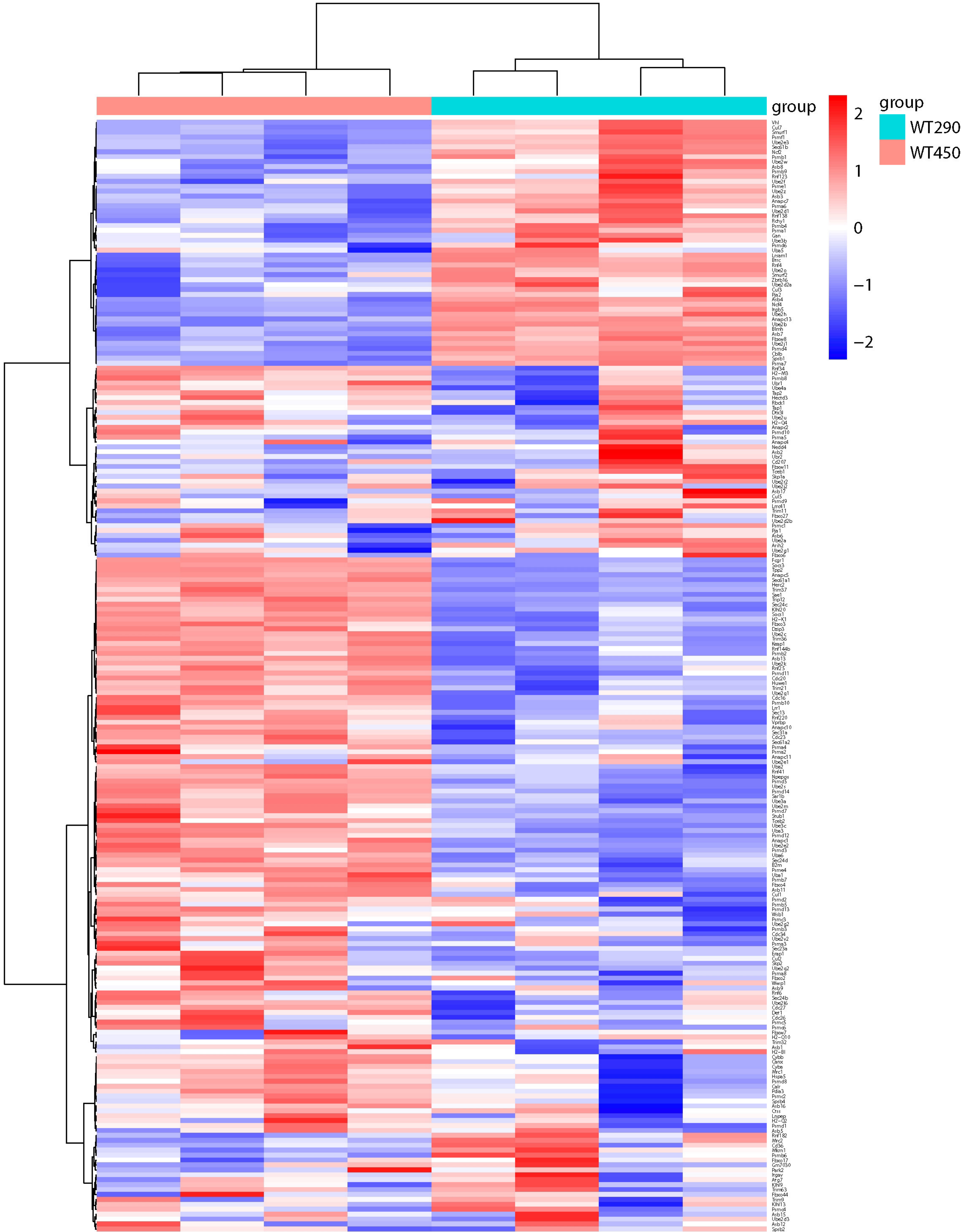

Supplementary Figure 6b.

CLASS_I_MHC_MEDIATED_ANTIGEN_PROCESSING_PRESENTATION

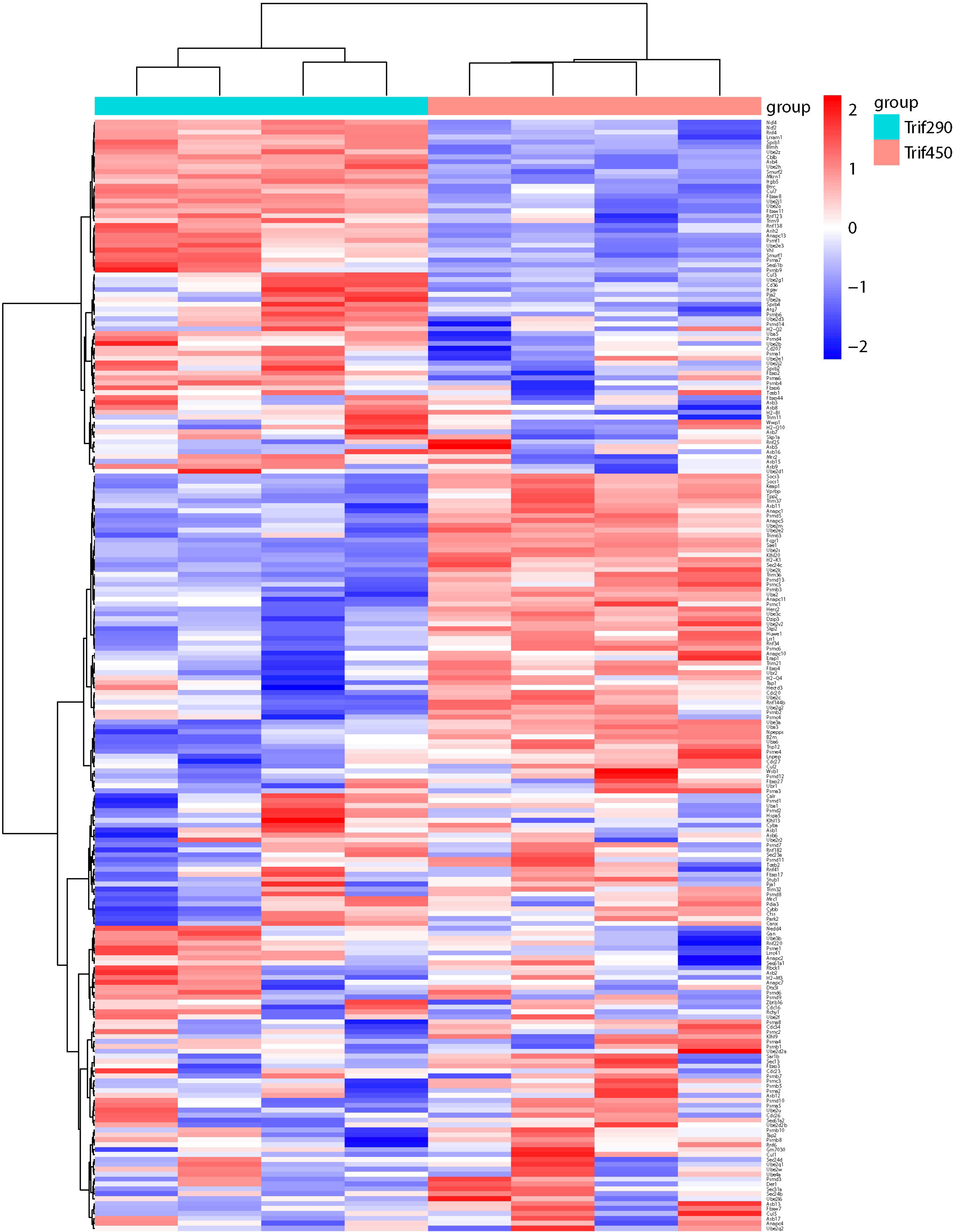

Supplementary table 1.

a)

Genes differentially regulated in WT(450 mOsm) vs WT (290 mOsm)			
Symbol	Gene name	logFC	adj. p value
Fcgr1	Fc receptor, IgG, high affinity I	0.99	7.66E-10
Socs3	suppressor of cytokine signaling 3	1.35	4.32E-08
Anapc5	anaphase-promoting complex subunit 5	0.60	1.19E-07
Sae1	SUMO1 activating enzyme subunit 1	0.52	8.81E-07
Herc2	HECT and RLD domain containing E3 ubiquitin protein ligase 2	0.40	7.22E-06
Psmd5	proteasome (prosome, macropain) 26S subunit, non-ATPase, 5	0.45	1.56E-05
Trim37	tripartite motif-containing 37	0.49	1.75E-05
Ube2c	ubiquitin-conjugating enzyme E2C	0.86	3.25E-05
Sec24c	Sec24 related gene family, member C (S. cerevisiae)	0.34	4.49E-05
Socs1	suppressor of cytokine signaling 1	0.60	6.11E-05
Uba6	ubiquitin-like modifier activating enzyme 6	0.42	8.07E-05
Ube2s	ubiquitin-conjugating enzyme E2S	0.33	0.00012
Ube3c	ubiquitin protein ligase E3C	0.34	0.000205
Rnf144b	ring finger protein 144B	0.52	0.000209
Cdc20	cell division cycle 20	0.74	0.000269
Anapc1	anaphase promoting complex subunit 1	0.25	0.000322
Ube3a	ubiquitin protein ligase E3A	0.36	0.000512
Asb13	ankyrin repeat and SOCS box-containing 13	0.44	0.000622
Asb11	ankyrin repeat and SOCS box-containing 11	0.42	0.000871
Uba3	ubiquitin-like modifier activating enzyme 3	0.28	0.000983
Keap1	kelch-like ECH-associated protein 1	0.26	0.00103
Klhl20	kelch-like 20	0.32	0.00121
Rnf34	ring finger protein 34	0.29	0.001575
Npepps	aminopeptidase puromycin sensitive	0.24	0.001636
Trim36	tripartite motif-containing 36	0.41	0.001837
Ube2e2	ubiquitin-conjugating enzyme E2E 2	0.32	0.001941
Tpp2	tripeptidyl peptidase II	0.22	0.002753
Sec61a1	Sec61 alpha 1 subunit (S. cerevisiae)	0.23	0.003246
Psmd14	proteasome (prosome, macropain) 26S subunit, non-ATPase, 14	0.21	0.003979
Ube2m	ubiquitin-conjugating enzyme E2M	0.21	0.004279
H2-K1	histocompatibility 2, K1, K region	0.22	0.004286
Trip12	thyroid hormone receptor interactor 12	0.19	0.004363
Ube2k	ubiquitin-conjugating enzyme E2K	0.21	0.004584
Fbxo3	F-box protein 3	0.21	0.004649
Cdc16	CDC16 cell division cycle 16	0.19	0.005579

Dzip3	DAZ interacting protein 3, zinc finger	0.30	0.007173
Lrr1	leucine rich repeat protein 1	0.46	0.007279
Psmb10	proteasome (prosome, macropain) subunit, beta type 10	0.22	0.007946
Psmb2	proteasome (prosome, macropain) subunit, beta type 2	0.22	0.009438
Skp2	S-phase kinase-associated protein 2 (p45)	0.25	0.012214
Psmd12	proteasome (prosome, macropain) 26S subunit, non-ATPase, 12	0.17	0.013455
Trim21	tripartite motif-containing 21	0.27	0.013652
Sec24d	Sec24 related gene family, member D (S. cerevisiae)	0.20	0.016178
Sec31a	Sec31 homolog A (S. cerevisiae)	0.17	0.019667
Sec61a2	Sec61, alpha subunit 2 (S. cerevisiae)	0.36	0.020686
Cybb	cytochrome b-245, beta polypeptide	0.27	0.02681
Uba2	ubiquitin-like modifier activating enzyme 2	0.17	0.029362
Ube2q2	ubiquitin-conjugating enzyme E2Q family member 2	0.18	0.030085
Sar1b	secretion associated Ras related GTPase 1B	0.18	0.030488
Huwe1	HECT, UBA and WWE domain containing 1	0.15	0.032962
B2m	beta-2 microglobulin	0.16	0.035251
Psme4	proteasome (prosome, macropain) activator subunit 4	0.16	0.035567
Fbxo4	F-box protein 4	0.20	0.036281
Rnf25	ring finger protein 25	0.16	0.037457
Rnf41	ring finger protein 41	0.16	0.038026
H2-M3	histocompatibility 2, M region locus 3	0.29	0.038432
Vprbp	Vpr (HIV-1) binding protein	0.27	0.039116
Ube2l6	ubiquitin-conjugating enzyme E2L 6	0.20	0.043067
Det1	de-etiolated homolog 1 (Arabidopsis)	0.20	0.045617
Psmd7	proteasome (prosome, macropain) 26S subunit, non-ATPase, 7	0.17	0.046887
Psmd11	proteasome (prosome, macropain) 26S subunit, non-ATPase, 11	0.14	0.049382
Rnf4	ring finger protein 4	-0.14	0.043094
Mkrn1	makorin, ring finger protein, 1	-0.14	0.04021
Rnf182	ring finger protein 182	-0.24	0.040198
Nedd4	neural precursor cell expressed, developmentally down-regulated 4	-0.39	0.039612
Psmb4	proteasome (prosome, macropain) subunit, beta type 4	-0.15	0.037309
Ube2z	ubiquitin-conjugating enzyme E2Z	-0.16	0.017921
Smurf2	SMAD specific E3 ubiquitin protein ligase 2	-0.22	0.01614
Ncf2	neutrophil cytosolic factor 2	-0.16	0.016036
Psmb9	proteasome (prosome, macropain) subunit, beta type 9 (large multifunctional peptidase 2)	-0.34	0.012199
Psma6	proteasome (prosome, macropain) subunit, alpha type 6	-0.19	0.011091
Psme1	proteasome (prosome, macropain) activator subunit 1 (PA28 alpha)	-0.22	0.00904
Anapc7	anaphase promoting complex subunit 7	-0.21	0.007511
Zbtb16	zinc finger and BTB domain containing 16	-0.38	0.004027
Ube2b	ubiquitin-conjugating enzyme E2B	-0.25	0.00223

Rnf138	ring finger protein 138	-0.62	0.001638
Sec61b	Sec61 beta subunit	-0.55	0.001194
Psmd4	proteasome (prosome, macropain) 26S subunit, non-ATPase, 4	-0.29	0.000787
Smurf1	SMAD specific E3 ubiquitin protein ligase 1	-0.47	0.000732
Asb7	ankyrin repeat and SOCS box-containing 7	-0.41	0.000525
Ube2e3	ubiquitin-conjugating enzyme E2E 3	-0.32	0.000456
Ube2o	ubiquitin-conjugating enzyme E2O	-0.32	0.000419
Asb3	ankyrin repeat and SOCS box-containing 3	-0.39	0.000359
Ube2j1	ubiquitin-conjugating enzyme E2J 1	-0.26	0.000358
Ube2h	ubiquitin-conjugating enzyme E2H	-0.39	0.000295
Psma7	proteasome (prosome, macropain) subunit, alpha type 7	-0.39	0.00019
Fbxw8	F-box and WD-40 domain protein 8	-0.33	0.000187
Btrc	beta-transducin repeat containing protein	-0.32	0.000177
Ncf4	neutrophil cytosolic factor 4	-0.42	0.000121
Lrsam1	leucine rich repeat and sterile alpha motif containing 1	-0.42	7.82E-05
Blmh	bleomycin hydrolase	-0.36	7.38E-05
Cul7	cullin 7	-0.59	7.06E-05
Vhl	von Hippel-Lindau tumor suppressor	-0.89	4.35E-05
Psmf1	proteasome (prosome, macropain) inhibitor subunit 1	-0.43	4.15E-05
Anapc13	anaphase promoting complex subunit 13	-0.40	2.78E-05
Itgb5	integrin beta 5	-1.05	2.16E-06
Spsb1	splA/ryanodine receptor domain and SOCS box containing 1	-0.94	1.57E-06
Asb4	ankyrin repeat and SOCS box-containing 4	-0.70	9.99E-07
Cblb	Casitas B-lineage lymphoma b	-1.46	8.82E-10

b)

**Upregulated in WT(450 mOsm) vs WT (290 mOsm), not upregulated in TRIF (450 mOsm) vs TRIF (290 mOsm)**			
Symbol	Gene name	Log-FC	adj. p value
Anapc1	anaphase promoting complex subunit 1	0.25	0.000322
Asb13	ankyrin repeat and SOCS box-containing 13	0.44	0.000622
Rnf34	ring finger protein 34	0.29	0.001575
Sec61a1	Sec61 alpha 1 subunit (S. cerevisiae)	0.23	0.003246
Psmd14	proteasome (prosome, macropain) 26S subunit, non-ATPase, 14	0.21	0.003979
Fbxo3	F-box protein 3	0.21	0.004649
Cdc16	CDC16 cell division cycle 16	0.19	0.005579
Psmb10	proteasome (prosome, macropain) subunit, beta type 10	0.22	0.007946
Psmb2	proteasome (prosome, macropain) subunit, beta type 2	0.22	0.009438
Psmd12	proteasome (prosome, macropain) 26S subunit, non-ATPase, 12	0.17	0.013455
Sec24d	Sec24 related gene family, member D (S. cerevisiae)	0.20	0.016178
Sec61a2	Sec61, alpha subunit 2 (S. cerevisiae)	0.36	0.020686

Cybb	Sec61, alpha subunit 2 (S. cerevisiae)	0.36	0.020686
Ube2q2	ubiquitin-conjugating enzyme E2Q family member 2	0.18	0.030085
Sar1b	secretion associated Ras related GTPase 1B	0.18	0.030488
Huwe1	HECT, UBA and WWE domain containing 1	0.15	0.032962
Fbxo4	F-box protein 4	0.20	0.036281
Rnf25	ring finger protein 25	0.16	0.037457
Rnf41	ring finger protein 41	0.16	0.038026
H2-M3	histocompatibility 2, M region locus 3	0.29	0.038432
Ube2l6	ubiquitin-conjugating enzyme E2L 6	0.20	0.043067
Det1	de-etiolated homolog 1 (Arabidopsis)	0.20	0.045617
Psmd7	proteasome (prosome, macropain) 26S subunit, non-ATPase, 7	0.17	0.046887
Psmd11	proteasome (prosome, macropain) 26S subunit, non-ATPase, 11	0.14	0.049382
**Downregulated in WT(450) vs WT (290), not downregulated in TRIF (450) vs TRIF (290)**			
Symbol	Gene name	Log-FC	adj. p value
Asb3	ankyrin repeat and SOCS box-containing 3	-0.39	0.000359
Psmd4	proteasome (prosome, macropain) 26S subunit, non-ATPase, 4	-0.29	0.000787
Ube2b	ubiquitin-conjugating enzyme E2B	-0.25	0.00223
Zbtb16	zinc finger and BTB domain containing 16	-0.38	0.004027
Anapc7	anaphase promoting complex subunit 7	-0.21	0.007511
Psme1	proteasome (prosome, macropain) activator subunit 1 (PA28 alpha)	-0.22	0.00904
Psma6	proteasome (prosome, macropain) subunit, alpha type 6	-0.19	0.011091
Psmb4	proteasome (prosome, macropain) subunit, beta type 4	-0.15	0.037309
Rnf182	ring finger protein 182	-0.24	0.040198
**Upregulated in TRIF (450) vs TRIF (290), not upregulated in WT(450) vs WT (290)**			
Symbol	Gene name	Log-FC	adj. p value
Psmd13	proteasome (prosome, macropain) 26S subunit, non-ATPase, 13	0.31	0.000444
Ube2v2	ubiquitin-conjugating enzyme E2 variant 2	0.26	0.007546
Anapc11	anaphase promoting complex subunit 11	0.23	0.012702
Psmb3	proteasome (prosome, macropain) subunit, beta type 3	0.20	0.015049
Psmc6	proteasome (prosome, macropain) 26S subunit, ATPase, 6	0.18	0.028856
Psmc5	protease (prosome, macropain) 26S subunit, ATPase 5	0.20	0.032952
**Downregulated in TRIF (450) vs TRIF (290), not downregulated in WT(450) vs WT (290)**			
Symbol	Gene name	Log-FC	adj. p value
Cul3	cullin 3	-0.21	0.011912
Fbxw11	F-box and WD-40 domain protein 11	-0.21	0.013861
Arih2	ariadne RBR E3 ubiquitin protein ligase 2	-0.21	0.018685
Psmb6	proteasome (prosome, macropain) subunit, beta type 6	-0.23	0.020911
Cd36	CD36 antigen	-0.50	0.032159
Ube2j2	ubiquitin-conjugating enzyme E2J 2	-0.19	0.048786
